# A Hox Transcription Factor Collective Binds a Highly Conserved *Distal-less* cis-Regulatory Module to Generate Robust Transcriptional Outcomes

**DOI:** 10.1371/journal.pgen.1005981

**Published:** 2016-04-08

**Authors:** Juli D. Uhl, Arya Zandvakili, Brian Gebelein

**Affiliations:** 1 Division of Developmental Biology, Cincinnati Children’s Hospital, Cincinnati, Ohio, United States of America; 2 Graduate Program in Molecular and Developmental Biology, Cincinnati Children's Hospital Research Foundation, Cincinnati, Ohio, United States of America; California Institute of Technology, UNITED STATES

## Abstract

*cis*-regulatory modules (CRMs) generate precise expression patterns by integrating numerous transcription factors (TFs). Surprisingly, CRMs that control essential gene patterns can differ greatly in conservation, suggesting distinct constraints on TF binding sites. Here, we show that a highly conserved *Distal-less* regulatory element (DCRE) that controls gene expression in leg precursor cells recruits multiple Hox, Extradenticle (Exd) and Homothorax (Hth) complexes to mediate dual outputs: thoracic activation and abdominal repression. Using reporter assays, we found that abdominal repression is particularly robust, as neither individual binding site mutations nor a DNA binding deficient Hth protein abolished cooperative DNA binding and *in vivo* repression. Moreover, a re-engineered DCRE containing a distinct configuration of Hox, Exd, and Hth sites also mediated abdominal Hox repression. However, the re-engineered DCRE failed to perform additional segment-specific functions such as thoracic activation. These findings are consistent with two emerging concepts in gene regulation: First, the abdominal Hox/Exd/Hth factors utilize protein-protein and protein-DNA interactions to form repression complexes on flexible combinations of sites, consistent with the TF collective model of CRM organization. Second, the conserved DCRE mediates multiple cell-type specific outputs, consistent with recent findings that pleiotropic CRMs are associated with conserved TF binding and added evolutionary constraints.

## Author Summary

Enhancers are regulatory elements that interact with transcription factor proteins to control cell-specific gene expression during development. Surprisingly, only a subset of enhancers are highly conserved at the sequence level, even though the expression patterns they control are often conserved and essential for proper development. Why some enhancer sequences are highly conserved whereas others are not is not well understood. In this study, we characterize a highly conserved enhancer that regulates gene expression in leg precursor cells. We find that this enhancer has dual regulatory activities that include gene activation in thoracic segments and gene repression in abdominal segments. Surprisingly, we show that the conserved enhancer can tolerate numerous sequence changes yet mediate robust transcription factor binding and abdominal repression. These findings are consistent with abdominal transcription factors binding numerous different configurations of binding sites. So, why is this enhancer highly conserved? We found that overlapping sequences within the enhancer also contribute to thoracic activation, suggesting the enhancer sequences are under added functional constraints. Altogether, our results provide new insights into why some enhancers are highly conserved at the sequence level while others can tolerate sequence changes.

## Introduction

The generation of cell-specific gene expression patterns during development is critical for proper morphogenesis. Gene expression at the transcriptional level is controlled by *cis*-regulatory modules (CRMs), which recruit transcription factor (TF) complexes that alter RNA polymerase activity [1–4]. In general, CRMs are relatively short genomic regions containing clustered binding sites for numerous sequence-specific TFs. CRM activity is determined by which TFs are expressed in each cell and the ability of these TFs to form active transcription complexes on CRM sequences [2,5]. Recently, large-scale genomic studies have identified thousands of CRMs [6–11]. Furthermore, human studies have increasingly found disease-associated single-nucleotide polymorphisms (SNPs) within putative CRMs [6–11]. Hence, understanding how CRMs integrate the appropriate combination of TFs to yield cell-specific transcriptional outcomes is fundamental to understanding both normal development and disease.

Two aspects of TF biology make it hard to predict CRM activity based on primary sequence. First, most TFs bind short degenerate DNA sequences present in high copy numbers throughout the genome [12]. Hence the number of potential genomic binding sites for a TF can exceed the number of TF molecules within a nucleus [13]. Second, the number of TFs encoded in the metazoan genome (>1000 in the human genome) makes predicting which specific TFs bind and regulate a CRM difficult [12]. For example, most TFs are members of large protein families that bind similar DNA sequences, yet CRMs are typically regulated by only one or a small subset of factors from each TF family [12]. Thus, the challenge lies in predicting which particular TFs will functionally bind which of the multitude of potential TF binding sites.

To better understand this problem, three models have been proposed for how CRMs integrate transcriptional inputs: the enhanceosome, the billboard, and the TF collective [5,14,15]. All three models require clustered TF binding sites, but they differ in both sequence conservation and modes of TF recruitment. Enhanceosomes are highly conserved, and recruit a highly cooperative TF complex. Known enhanceosomes have rigid constraints on the order, spacing, and orientation of binding sites, and point mutations in single sites disrupt both complex formation and transcriptional output. The best-characterized enhanceosome is the interferon-β enhancer that coordinates the stepwise recruitment of a series of TFs to mediate high levels of transcriptional activation following viral infection [15,16]. In contrast, billboard CRMs are characterized by flexible orientations/spacing of binding sites that recruit TFs independently and are thereby under less evolutionary constraint [14,17]. The rapid evolution and rearrangement of binding sites within the *even-skipped* (*eve*) stripe 2 enhancer in dipterans supports the flexible billboard model [18–21]. The TF collective model proposes that groups of TFs form cooperative complexes on CRMs via a combination of protein-DNA and protein-protein interactions [5]. Unlike the enhanceosome, however, the TF collective posits that protein-protein interactions provide flexibility that eases binding site constraints. For example a TF can be recruited to CRMs lacking its binding site as long as there are sufficient sites for the other TFs of the collective. A collective of five TFs form transcription complexes on numerous CRMs containing various combinations of TF binding sites to regulate gene expression in the *Drosophila* heart [22].

The differing requirements for how TF sites are organized between the enhanceosome, billboard, and TF collective models may help explain the varying degree of sequence conservation between CRMs. Genomic sequencing of related species revealed that only a subset of CRMs involved in regulating developmentally important genes are highly conserved [23]. For example, the *Drosophila vestigial* boundary enhancer contains blocks of high sequence conservation, while the *eve* stripe 2 enhancer is not highly conserved at the sequence level [19,24,25]. This raises an interesting question; why are only some developmentally important CRMs highly conserved? While the answer is currently unclear, one reason may be the different ways CRMs integrate TFs. The enhanceosome model requires tight constraints on TF binding sites consistent with high sequence conservation. In contrast the billboard and TF collective models relax constraints on binding sites, consistent with rapid sequence turnover. Unfortunately, few highly conserved CRMs have been thoroughly dissected and thus, we lack an understanding of which models best explain CRM function and conservation.

**Fig 1:**
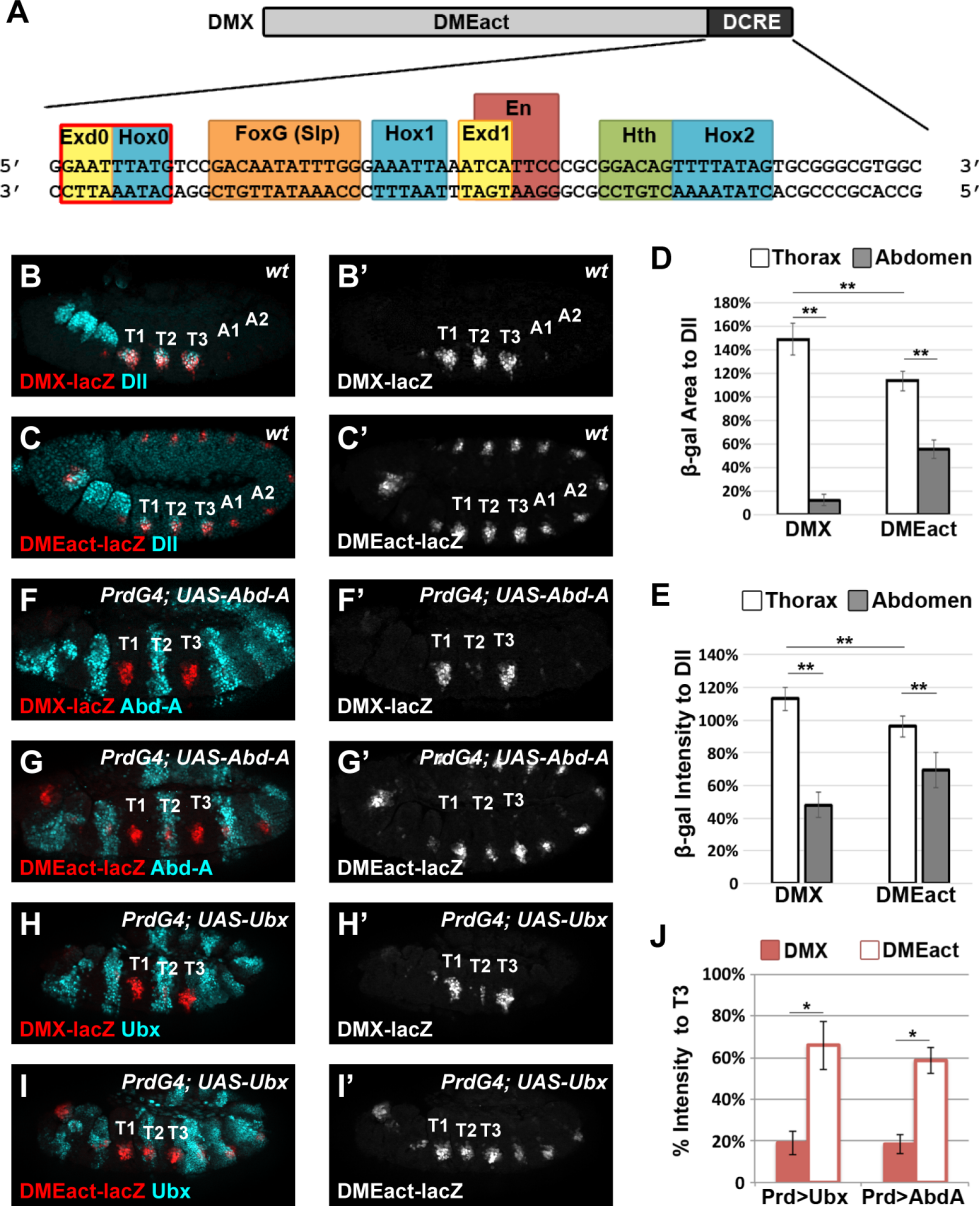
The abdominal Hox factors repress *Distal-less* via DCRE-dependent and–independent mechanisms. (A) Schematic of the DMX enhancer containing the DMEact and the DCRE. Detail shows the DCRE sequence with known TF binding sites (FoxG, Hox1, Exd, En, Hth, and Hox2) highlighted. Note, the Exd0 and Hox0 sites are new sites characterized in this manuscript. (B) *DMX-lacZ* is expressed in Dll-positive (Dll+) cells of the thorax. (C) *DMEact-lacZ* is expressed in Dll+ cells of the thorax and the corresponding cells of the abdomen. (D-E) Quantification of β-gal expression area (D) and β-gal intensity relative to Dll expression in the same embryos (E) in *DMX-lacZ* and *DMEact-lacZ* embryos demonstrates that the DMEact is not fully de-repressed in the abdomen and has reduced thoracic levels when compared to thoracic levels of DMX. (F-I) Effect of Hox gene mis-expression on reporter activity in *PrdG4;UAS-AbdA;DMX-lacZ* (F), *PrdG4;UAS-AbdA;DMEact-lacZ* (G), *PrdG4;UAS-Ubx;DMX-lacZ* (H), and *PrdG4;UAS-Ubx;DMEact-lacZ* (I) embryos. (J) Quantification of β-gal levels in T2 mis-expressing segments relative to non-Gal4 expressing control T3 segments shows that both Abd-A and Ubx repress via DCRE-dependent and -independent mechanisms. All images are lateral views of Stage 11 embryos immunostained for β-gal (red or white) and Dll, Abd-A, or Ubx (cyan) as indicated. (Statistics ** p < 0.01, Welch’s t-test, error bars S.E.M). Note, as a control for the PrdG4 experiments, we quantified the levels of βgal in the absence of Gal4 and noted no significant differences between the T2 vs T3 segments or the A1 vs A2 segments of *DMX-lacZ* and *DMEact-lacZ* embryos. (DMX β-gal pixel intensity: Thorax, T2 = 113 ± 13%, T3 = 112 ± 10%, Abdomen, A1 = 44 ±10%, A2 = 56 ± 13%. DMEact β-gal pixel intensity: Thorax, T2 = 98 ± 12%, T3 = 96 ± 11, Abdomen, A1 = 69 ±12%, A2 = 70 ± 16%).

The DMX is a conserved CRM that activates the *Distal-less* (*Dll*) appendage selector gene in thoracic segments to initiate leg development [26–28]. While activators that can stimulate the DMX are also present in the abdomen, DMX activity is restricted to the thorax via a highly conserved sequence (the *Distal-less* conserved regulatory element, DCRE) [27,28]. Previous studies demonstrated that the DCRE represses transcription by recruiting TF complexes containing abdominal Hox factors (either Ultrabithorax (Ubx) or Abdominal-A (Abd-A)), Extradenticle (Exd), Homothorax (Hth), Engrailed (En), and the FoxG Sloppy-paired TF (Slp1 and Slp2, referred to here as Slp) [28,29] (Fig 1A). Like the Hox factors, Exd (vertebrate Pbx) and Hth (vertebrate Meis) are conserved homeodomain TFs that regulate segment identity and cell fates along the anterior-posterior axis of metazoans [30–32]. Exd and Hth form cooperative TF complexes with Hox factors on DNA via several protein-protein interactions, and the DCRE recruits an abdominal Hox/Exd/Hth/Hox complex via two Hox binding sites (Hox1 and Hox2) that are coupled to either adjacent Exd (Exd1) or Hth sites (Fig 1A). DCRE-mediated repression also requires compartment-specific inputs with an En site needed for posterior-compartment repression, and FoxG (Slp) sites are required for anterior-compartment repression (Fig 1A and [28]).

Based on the presence of high sequence conservation, one may reasonably predict that a highly conserved CRM such as the DCRE indicates constrained interactions between TFs as in the enhanceosome model. Here we provide evidence that despite high sequence conservation, the DCRE is most consistent with the TF collective model of CRM function. First, we used quantitative transgenic reporter and DNA binding assays to show that the DCRE contains an additional Exd/Hox site (Exd0/Hox0, Fig 1A), and that multiple combinations/configurations of linked Hox/cofactor binding sites can mediate robust transcriptional repression. Unlike the independent TF binding of the billboard model, however, we found that abdominal Hox, Exd, and Hth factors mediate cooperative TF complex formation on the DCRE. Moreover, cooperative complex formation and transcriptional repression can tolerate both individual DNA binding site mutations as well as deletion of the Hth DNA binding domain. These findings are consistent with the TF collective model of CRM function. However, we also found that the linked Hox/cofactor sites in the DCRE enhance thoracic *Dll* expression in a Hox-dependent manner, and that the re-configured Hox/cofactor binding sites failed to perform all DCRE-dependent functions. Taken together, these findings suggest that the pleiotropic functions of the DCRE (thoracic activation and abdominal repression) add constraints that limit sequence variation, thus providing a potential mechanistic understanding for why some CRMs are highly conserved.

## Results

### The abdominal Hox factors repress *Distal-less* via DCRE-dependent and -independent mechanisms

Thoracic *Distal-less* (*Dll*) expression is essential for the specification of leg precursor cells of the *Drosophila* embryo [26,33]. Previous studies identified a conserved *Dll* CRM, the DMX, which mediates early thoracic leg expression [26]. DMX contains two distinct regions: the DMEact (bp 1–661), which activates gene expression in thoracic and abdominal segments, and an abdominal repression element (Fig 1A) [26,28,34]. The repression element has been defined several times based on different criteria including restriction enzyme sites (“NRE-BX” bp 681–877 [26]), functional studies (“DllR” bp 681–713 [27]), and genomic conservation (“DMXR” bp 675–731 [28]). In this study, we use conservation across 21 *Drosophila* species to define the repression element as the Distal-less Conserved Regulatory Element (DCRE), bp 662–731, (S1 Fig). This conserved sequence contains six previously characterized TF binding sites, including the linked Hox1/Exd1 and Hth/Hox2 sites that recruit a cooperative abdominal Hox complex as well as FoxG (Slp) and En binding sites, all of which are required for complete abdominal segment repression (Fig 1A and [26,28,35]).

Our current understanding of DMX function suggests the DMEact (1–661) mediates equal activation in all body segments (thorax and abdomen) and the DCRE (662–731) mediates abdominal repression to restrict expression to the thorax. To test these ideas, we integrated *DMEact-lacZ* (DCRE-lacking) and *DMX-lacZ* (DCRE-containing) into the same genomic locus and measured β-gal expression normalized to thoracic Dll expression in age-matched embryos. If the DCRE only contributes to abdominal repression, then *DMEact-lacZ* and *DMX-lacZ* embryos should have equal levels of thoracic expression. However, the DCRE-lacking *DMEact-lacZ* embryos express β-gal in significantly fewer thoracic cells, and those that do, express β-gal at lower levels when compared to *DMX-lacZ* embryos (Fig 1B–1E). Next, we determined if the DMEact is capable of equal activation in thoracic and abdominal segments in the absence of the DCRE by comparing thoracic versus abdominal gene expression in *DMEact-lacZ* embryos. We found significantly fewer abdominal cells express β-gal and those that do have reduced levels compared to thoracic cells (Fig 1C–1E). Taken together, these findings show that the DMEact and DCRE each contribute to thoracic and abdominal gene regulation, and together yield expression differences between the thorax and abdomen.

Because thoracic and abdominal *DMEact-lacZ* levels differ, we hypothesized that abdominal Hox factors repress the DMX in a DCRE-independent manner. To test this idea, we mis-expressed Abd-A or Ubx using *Paired-Gal4* (*PrdG4*) and measured *DMX-lacZ* and *DMEact-lacZ* activity in the thorax. *PrdG4* is active in every other segment, which allows for direct comparisons between experimental (T2) and wild type segments (T1/T3). Care was taken to use conditions that express near physiological levels of Ubx and Abd-A (see Materials and Methods). As expected, either Ubx or Abd-A repressed approximately 80% of *DMX-lacZ* activity in experimental (T2) segments relative to control T3 segments (Fig 1F and 1H and 1J). Importantly, either also repressed *DMEact-lacZ*, though to a lesser extent than *DMX-lacZ* (~40%, Fig 1G and 1I and 1J), indicating that Hox factors repress the DMEact either through direct binding or indirectly through the repression of thoracic activators. Thus, abdominal Hox factors repress the DMX through DCRE-dependent and DCRE-independent mechanisms.

### The DCRE mediates two transcriptional outcomes: Abdominal repression in Sloppy-paired-positive cells and conditional thoracic activation

**Fig 2:**
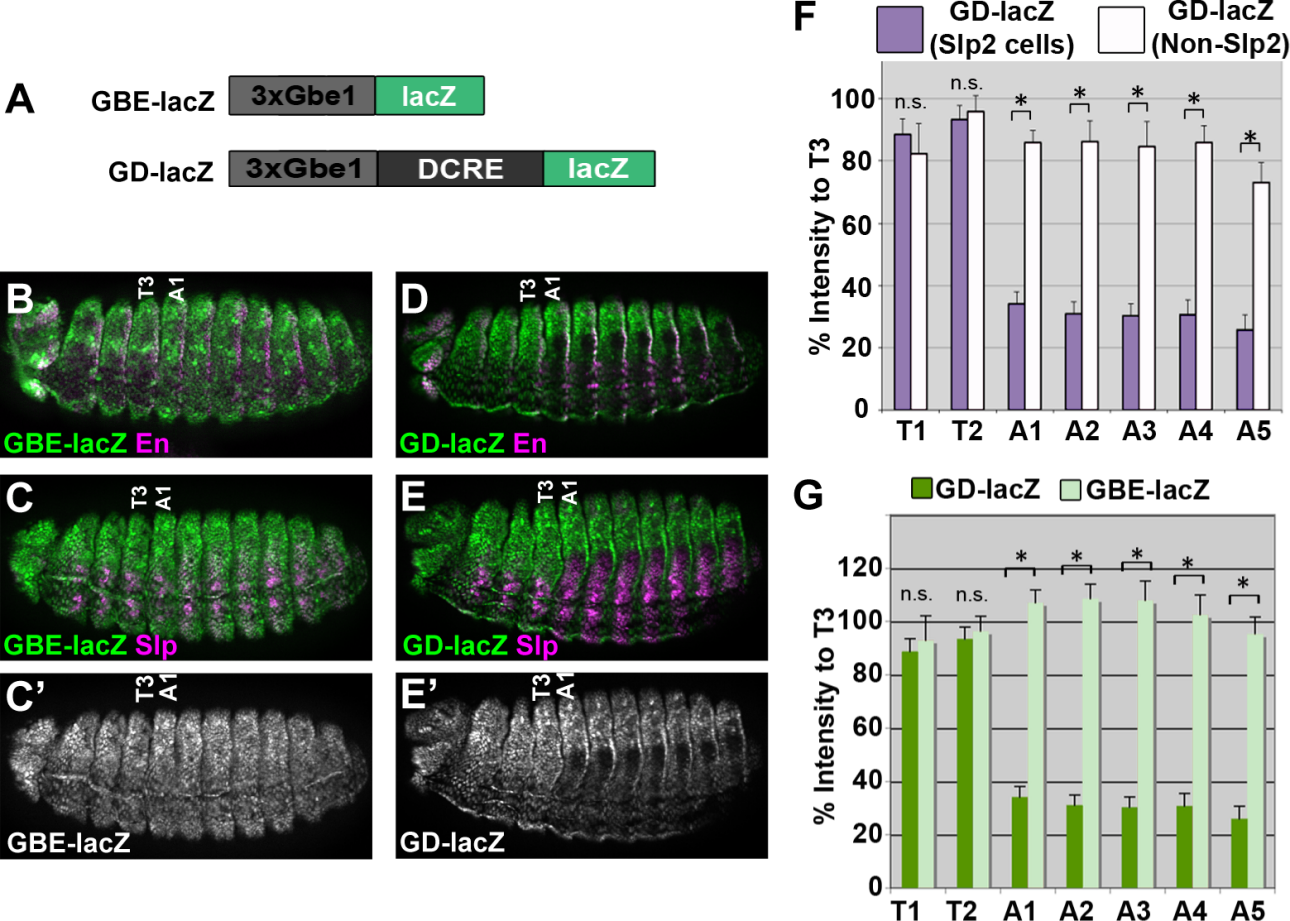
The DCRE is sufficient for repression in a cell-specific manner in the abdomen. (A) Schematics of the *GBE-lacZ* and *GD-lacZ* reporter assays. (B, C) *GBE-lacZ* embryos reveal relatively uniform β-gal expression in posterior En+ cells (B) and anterior Slp2+cells (C). (D, E) *GD-lacZ* embryos reveal that the DCRE represses gene expression in Slp2+ (E) but not En+ (D) abdominal cells. (F) Quantification of β-gal intensity of thoracic and abdominal segments relative to segment T3 (see Materials and Methods) for *GD-lacZ* activity in Slp2+ versus Slp2- cells demonstrates that the DCRE represses gene expression in Slp+ cells of the abdomen. (G) Quantification of β-gal intensity of thoracic and abdominal segments of *GD-lacZ* versus *GBE-lacZ*. All images are lateral views of Stage 15 embryos immunostained for β-gal (green or white) and Slp2 or En (magenta) as indicated. (Statistics * p < 0.05, n.s. not significant, Welch’s t-test, Error Bars S.E.M).

To better characterize Hox-mediated regulation of the DCRE, we generated two synthetic transgenic reporter assays to isolate the DCRE from other DMX regulatory sequences. First, we created an abdominal repression assay by placing *lacZ* under the control of three copies of the Grainyhead-binding element 1 (*GBE-lacZ*) (Fig 2A). Embryos containing *GBE-lacZ* exhibit strong uniform epidermal expression during stage 15 [36] (Fig 2B and 2C). Incorporating the DCRE (*GD-lacZ*) resulted in a pronounced decrease in β-gal expression within a subset of abdominal cells compared to *GBE-lacZ* embryos (Fig 2B–2E). Previous studies showed that the DCRE mediates repression in a compartment-specific manner within the context of the DMX enhancer [28]. In the posterior compartment, abdominal Hox factors repress with Engrailed (En), whereas in the anterior compartment they repress with the FoxG factors, Sloppy-paired (Slp1 and Slp2). In the *GD-lacZ* assay, the DCRE is sufficient to repress transcription within abdominal cells that express Slp (Fig 2E). However, the DCRE is not sufficient for posterior compartment repression, suggesting that En and Hox repression through the DCRE requires additional sites within the DMEact. Quantification of β-gal levels in Slp2+ cells of *GD-lacZ* embryos revealed a 70% decrease in abdominal segments relative to thoracic segments, whereas β-gal levels were equivalent between Slp2+ thoracic cells and Slp2-negative thoracic and abdominal cells (Fig 2F). Importantly, repression in Slp2+ cells is DCRE-dependent as no difference in β-gal was observed between thoracic and abdominal Slp2+ cells in *GBE-lacZ* embryos (Fig 2G). Thus, *GD-lacZ* is a quantifiable assay to study the mechanisms of DCRE-mediated abdominal repression in Slp+ cells.

**Fig 3:**
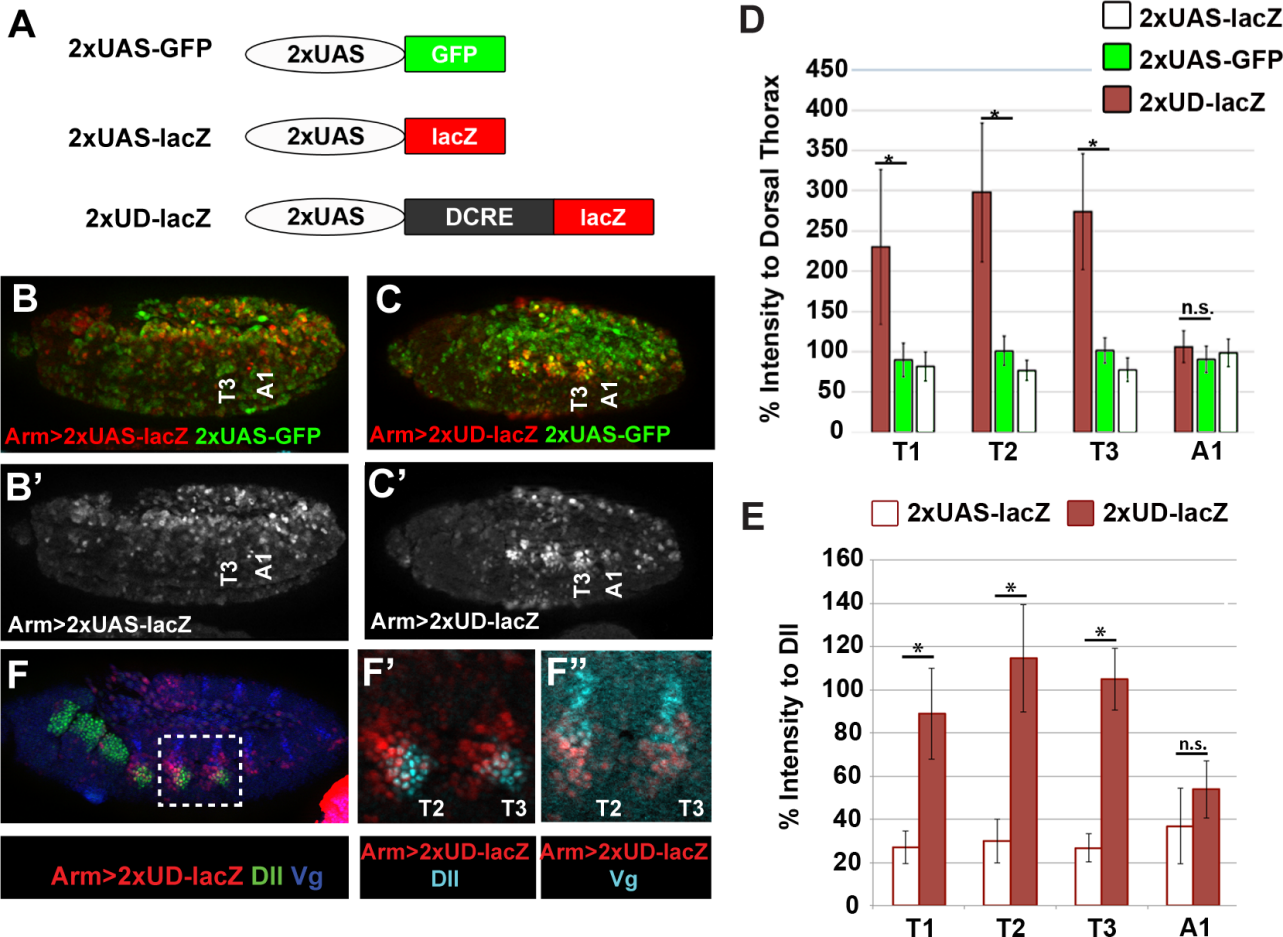
The DCRE is a conditional activator in the thorax. (A) Schematics of *2xUAS-lacZ*, *2xUAS-GFP* and *2xUD-lacZ* reporter assays. (B) *Armadillo-Gal4* driving expression of *2xUAS-lacZ* and *2xUAS-GFP* reveals stochastic reporter expression in the epidermis of Stage 11 embryos immunostained for β-gal (red/white) and GFP (green). (C) *Armadillo-Gal4* driving expression of *2xUD-lacZ* and *2xUAS-GFP* reveal that the DCRE selectively enhances transcription in thoracic segments of Stage 11 embryos. (D) Quantification of β-gal intensity relative to dorsal thorax (T1) of *2xUAS-lacZ* or *2xUD-lacZ* and GFP intensity relative to dorsal thorax (T1) of *2xUAS-GFP* embryos imaged under identical conditions reveals that though stochastic, the 2xUAS sites drive similar reporter expression in the abdomen and thorax (ANOVA of *2xUAS-lacZ* in T1, T2, T3, A1, p = 0.25, ANOVA of *2xUAS-GFP* in T1, T2, T3, A1, p = 0.69). The *2xUD-lacZ* drives significantly higher reporter expression in the thorax compared to either the abdomen or the *2xUAS-lacZ* or *2xUAS-GFP* reporters in the same segment. (Statistics comparing 2xUD-lacZ to 2xUAS-GFP in same embryo by segment, *p<0.05, n.s. not significant, Welch’s t-test, error bars are S.E.M.) (E) Quantification of β-gal intensity relative to Dll intensity in *2xUAS-lacZ* and *2xUD-lacZ* embryos reveals the DCRE significantly increases thoracic but not abdominal expression. (*p<0.05, Welch’s t-test, error bars are S.E.M. (F) Lateral view of a *2xUD-lacZ* embryo immunostained for β-gal (red), Dll (green or cyan as indicated), and Vg (blue or cyan as indicated) demonstrates that most *2xUD-lacZ* activity is located in Dll+ or Vg+ cells.

The second synthetic reporter assay consists of *lacZ* under control of two copies of the upstream activation sequence (UAS) that can be activated by Gal4 (*2xUAS-lacZ*) (Fig 3A). When *2xUAS-lacZ* is crossed to ubiquitous Gal4 drivers such as *armadillo-Gal4* (*ArmG4)*, relatively weak, stochastic expression is observed in stage 11 embryos (Fig 3B). Incorporating the DCRE into the 2xUAS reporter (*2xUD-lacZ*) and crossing to *ArmG4* surprisingly did not reveal abdominal repression, suggesting the DCRE cannot repress Gal4-mediated activation (Fig 3C and 3D and 3E). However, consistent with the DCRE enhancing thoracic expression in the context of the DMX, analysis of *2xUD-lacZ* activity in the thorax revealed a 2 to 3 fold increase in β-gal levels relative to control *2XUAS-lacZ* embryos (Fig 3B and 3C and 3E). Note, we also observed enhanced thoracic expression relative to abdominal segments in early *GD-lacZ* embryos, but this difference is lost in older embryos due to the uniform increase in strength of the grainy-head activator (compare thoracic reporter activity in Slp2+ and Slp2- cells in Fig 2F). To better quantify the effect the DCRE has on thoracic gene expression in the UAS assay, we incorporated a control *2xUAS-GFP* reporter and found that while *2xUAS-GFP* and *2xUAS-lacZ* are both expressed stochastically, the relative levels of the two reporters are equal between the thorax and abdomen (Fig 3B–3D). In contrast, β-gal expression from *2xUD-lacZ* is significantly increased relative to *2xUAS-GFP* expression in thoracic but not abdominal cells (Fig 3D and 3E). A similar induction was observed using different drivers (*Tubulin-Gal4*, *Daughterless-Gal4)* yet no expression was observed in *2XUD-lacZ* embryos lacking a Gal4 driver (S2 Fig). Hence, the DCRE is insufficient to initiate gene expression on its own, but it can selectively enhance transcription in thoracic segments.

Like abdominal repression in the *GD-lacZ* assay, enhanced thoracic activation of *2xUD-lacZ* was observed in only a subset of cells, even though *ArmG4* is active throughout these segments as shown by *2xUAS-GFP* expression (Fig 3C and 3D). Co-stains revealed that enhanced β-gal largely overlaps with Dll+ cells and a group of Vestigial (Vg)-positive cells that arise from the Dll+ leg primordia (Fig 3F) [33,37]. These results are consistent with the finding that the DCRE-containing *DMX-lacZ* expresses significantly higher β-gal in Dll+ cells of the thorax than the DCRE-lacking *DMEact-lacZ* (Fig 1D and 1E).

Altogether, these results support a model whereby the DCRE mediates multiple cell-specific transcriptional outputs: In the abdomen, the DCRE is sufficient to repress transcription in a cell-specific manner (Slp+ cells) in the anterior compartment. In addition, the DCRE contributes to abdominal repression in the posterior compartment in the context of the DMX [28], but the DCRE is not sufficient to perform this function in isolation from the other DMX sequences. In the thorax, the DCRE functions as a conditional activation element that does not initiate expression but can increase transcription of both endogenous (*DMEact*) and heterologous (*2xUAS*) enhancers in the leg primordia. Thus, the *GD-lacZ* and *UD-lacZ* assays provide tools that can be used to study the role of Hox, Exd, and Hth factors in regulating a subset of DCRE-mediated activities in isolation from the other DMX regulatory sequences.

### Homothorax and Sloppy-paired are required for DCRE-mediated repression

**Fig 4:**
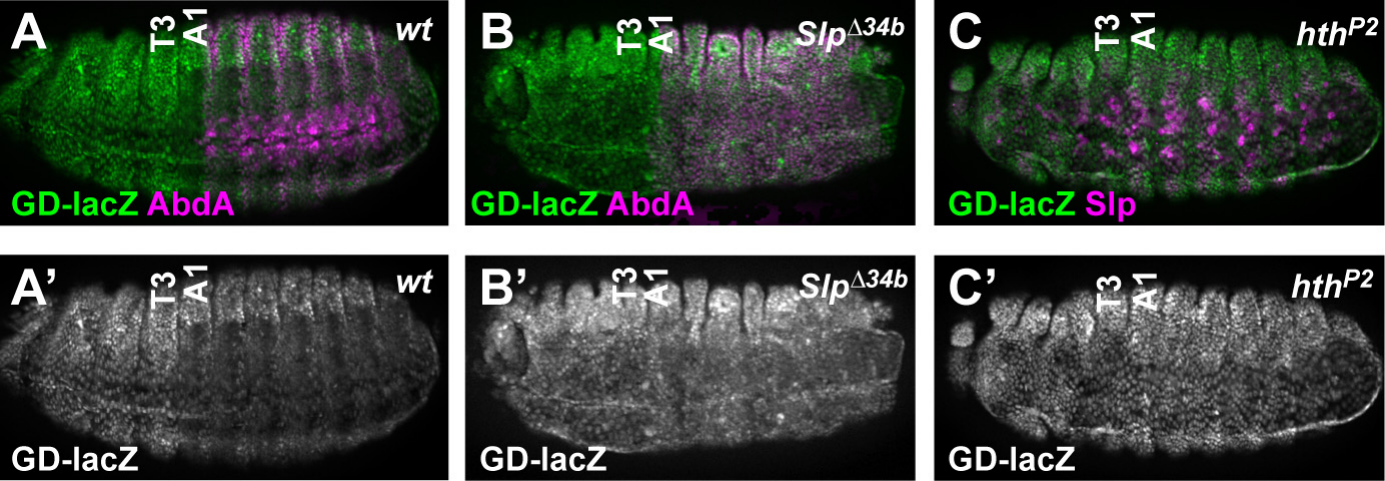
Homothorax and Sloppy-paired are genetically required for DCRE-mediated repression. (A-C) *GD-lacZ* activity in *wt* (A), *Slp*^*Δ34*^ (B) and *hth*^*P2*^ (C) embryos reveals that Slp and Hth are required for DCRE-repression. All images are lateral views of Stage 15 embryos immunostained for β-gal (green or white) and AbdA or Slp (magenta) as indicated.

The published model of DCRE-mediated repression in the anterior compartment requires an abdominal Hox factor (Ubx or Abd-A), the Exd and Hth cofactors, and a FoxG Slp factor [26,28]. However, genetic removal of *hth*, *exd*, or *Slp* results in severe embryonic defects, including the loss of *wingless* (*wg*) expression, which is required for DMX activation [33,38,39]. Since *GD-lacZ* does not require Wg for activation, it provides a useful tool for genetic tests of these factors. While a deletion removing both *Slp1* and *Slp2* (*Slp*^*∆34b*^) results in gross morphological abnormalities due to segmentation defects [29], *GD-lacZ* expression is equal in the thorax and abdomen of Slp mutant embryos (Fig 4A and 4B). Thus, Slp factors are required to mediate DCRE repression. To assess the roles of Hth and Exd, we took advantage of the finding that *hth* and *exd* are co-dependent for proper function; genetic removal of *hth* results in exclusion of Exd protein from the nucleus [30,40,41]. Hence, we assayed *GD-lacZ* activity in a severe hypomorph of *hth* (*hth*^*P2*^) and found abdominal repression is abolished (Fig 4C). Since abdominal Hox factors are expressed in both *Slp* and *hth* mutant embryos [40,42], these findings demonstrate abdominal Hox factors are insufficient to mediate DCRE repression. However, at least one abdominal Hox factor is required for repression. *GD-lacZ* activity in single *Ubx*^*1*^ and *Abd-A*^*M1*^, and double *Ubx*^*1*^*Abd-A*^*MX1*^ null embryos revealed that either abdominal Hox factor mediates DCRE-repression whereas removal of both abolishes repression (S3 Fig). Together, these data support the model that the DCRE integrates abdominal Hox/Exd/Hth complexes with the Slp FoxG factors to repress abdominal gene expression.

### DCRE-mediated thoracic activation requires Antennapedia and Hth

**Fig 5:**
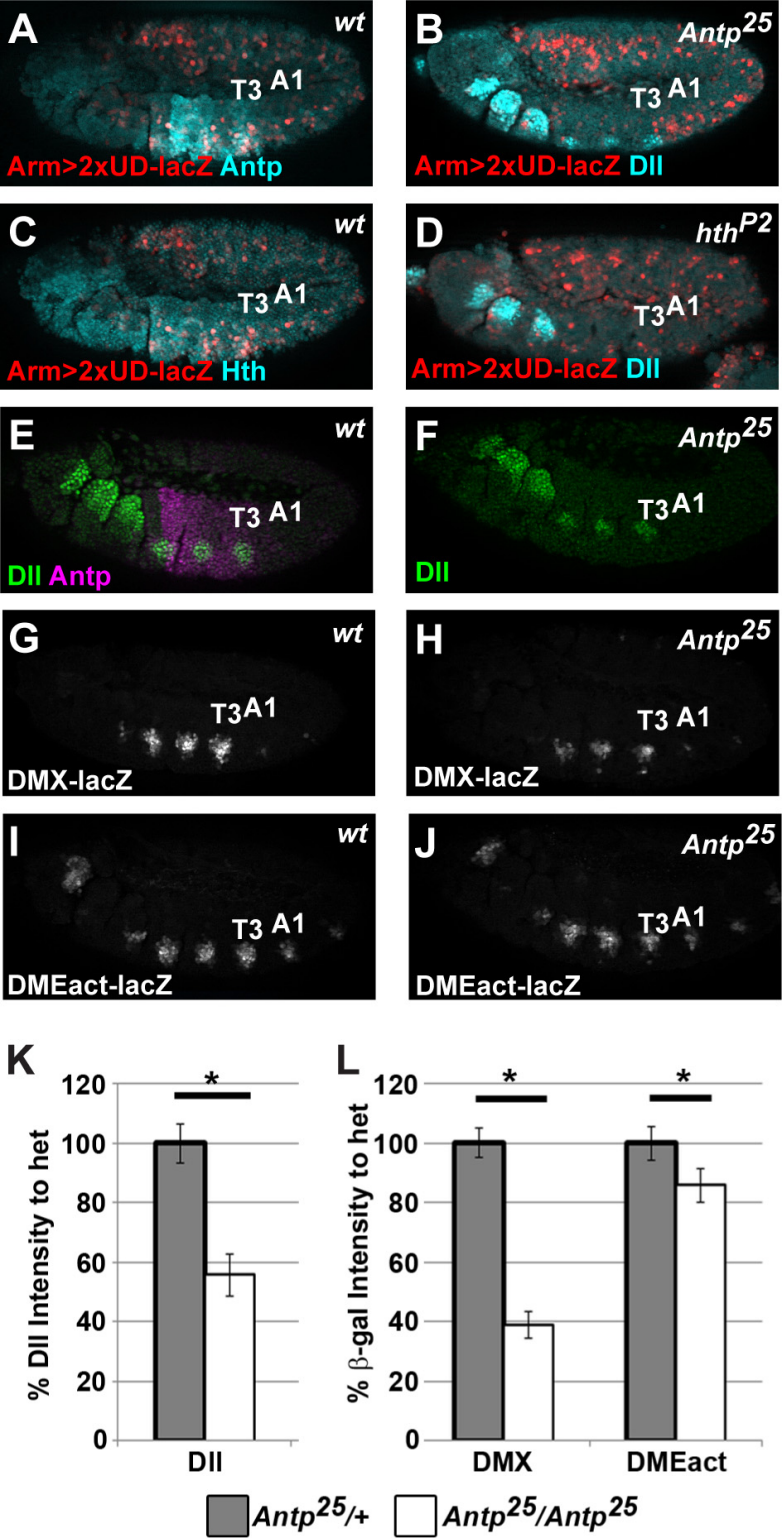
Antp and Hth are required for DCRE-mediated thoracic activation. (A) *ArmG4;2xUD-lacZ* embryo immunostained for Antp and β-gal demonstrates that DCRE-mediated thoracic activation is found in Antp+ cells. (B) *Antp*^*25*^ null embryo containing *ArmG4;2xUD-lacZ* demonstrates thoracic activation requires the Antp Hox factor. (C) *ArmG4;2xUD-lacZ* embryo immunostained for Hth and β-gal reveals significant co-expression. (D) Hth hypomorph (*Hth*^*P2*^) embryo containing *ArmG4;2xUD-lacZ* demonstrates that thoracic activation requires the Hth transcription factor. (E) Wild-type *(Antp25/+)* embryo immunostained for Dll and Antp. (F) *Antp*^*25*^/*Antp*^*25*^ null embryo immunostained for Dll reveals a reduction in Dll expression in the thorax. (G) *Antp*^*25*^*/+*;*DMX-lacZ* embryo immunostained for β-gal. (H) *Antp*^*25*^*/Antp*^*25*^ embryo containing *DMX-lacZ* reveals a substantial reduction in thoracic β-gal expression compared to the heterozygote. (I) *Antp*^*25*^*/+*;*DMEact-lacZ* embryo immunostained for β-gal. (J) *Antp*^*25*^*/Antp*^*25*^ embryo containing *DMEact-lacZ* reveals a slight reduction of thoracic β-gal expression compared to the heterozygote. (K) Quantification of Dll expression in *Antp*^*25*^*/ Antp*^*25*^ embryos relative to identically treated sibling *Antp*^*25*^*/+* embryos reveals that removal of Antp causes a significant reduction in Dll expression. (L) Quantification of β-gal expression from DMX or DMEact in *Antp*^*25*^*/ Antp*^*25*^ embryos compared to identically treated sibling *Antp*^*25*^*/+* embryos reveals that *DMX-lacZ* expresses substantially less when Antp is removed, while *DMEact-lacZ* is only slightly affected by Antp removal. All images are lateral views of Stage 11 embryos immunostained for β-gal (red or white), Antp (magenta), and Hth, or Dll (cyan or green) as indicated. (Statistics, * p<0.05, Welch’s t-test).

While a role for abdominal Hox factors in repressing *Dll* was previously established [26], no prior studies revealed a role for a thoracic Hox factor in activating *Dll*. The best candidate for a potential positive regulator of *Dll* is the Antennapedia (Antp) Hox factor, as Antp and nuclear Exd/Hth are co-expressed with Dll in thoracic cells that activate *2XUD-lacZ* (Fig 5A and 5C and 5E). Moreover, the enhanced thoracic β-gal expression of *2XUD-lacZ* is nearly eliminated in *Antp*^*25*^ null embryos as well as in *Hth*^*P2*^ embryos that lack both Hth and nuclear Exd (Fig 5B and 5D). These data suggest Antp directly contributes to thoracic *Dll* expression through the DCRE. To test this idea, we quantified Dll expression in *Antp*^*25*^ null mutants and heterozygous siblings and found a significant reduction of Dll levels (~40%, Fig 5E and 5F and 5K). In addition, we analyzed expression of *DMX-lacZ* and *DMEact-lacZ* in *Antp*^*25*^ mutants and found that the DCRE-containing DMX reporter lost over 50% of its thoracic activity in *Antp*^*25*^ null embryos whereas the DCRE-lacking DMEact reporter was not substantially different from heterozygous siblings (Fig 5G–5J and 5L). These data are consistent with Antp increasing *DMX-lacZ e*xpression levels in a DCRE-dependent manner.

### DCRE activity is mediated through linked Hox-Exd and Hox-Hth sites

**Fig 6:**
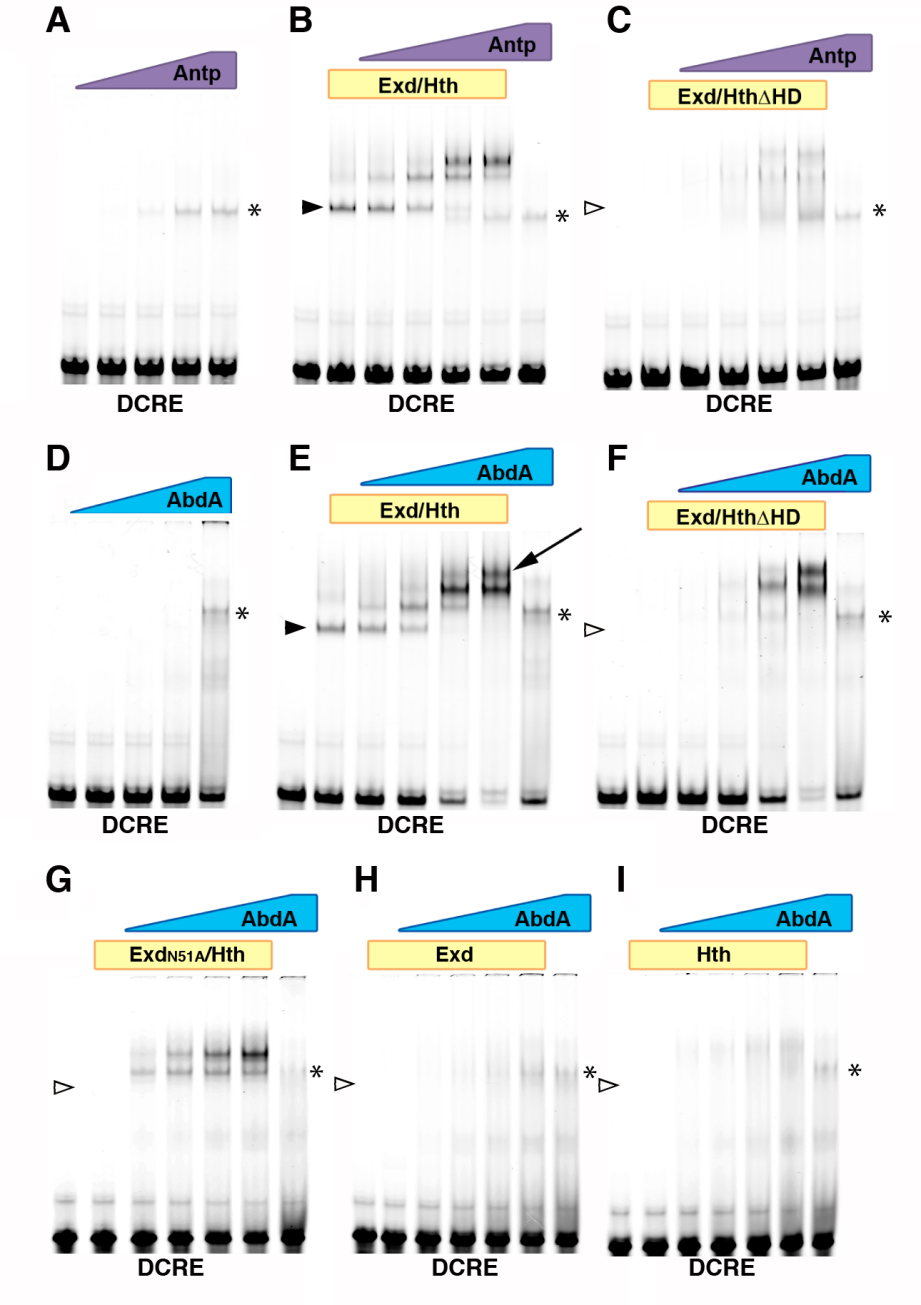
Hox binding to linked Hox/Exd and Hox/Hth sites within the DCRE. (A-I) EMSAs performed on the DCRE full-length probe (for sequence, see Fig 1A). (A-C) Titration of Antp protein (concentrations from 37.5 nM to 300 nM) alone (A), with 25 nM purified Exd/Hth dimer (B), or with 200 nM purified Exd/HthΔHD dimer (C). (D-I) Titration of AbdA protein (concentrations from 37.5 nM to 300 nM) on the DCRE wild-type probe alone or with (D) 75 nM purified Exd/Hth dimer (E) 200 nM purified Exd/HthΔHD dimer (F), 200nM purified ExdN51A/Hth (G), 200 nM purified Exd (H), or 200 nM purified Hth (I). * indicates Hox-only binding to DCRE. Arrow indicates higher order complex seen with AbdA/Exd/Hth but not Antp/Exd/Hth. Filled arrowheads indicate Hth/Exd heterodimer binding. Empty arrowheads indicate lack of binding by Hth and/or Exd.

The behavior of the DCRE in the *GD-lacZ*, *UD-lacZ* and *DMX-lacZ* reporters supports the idea that the DCRE conveys multiple transcriptional outcomes: thoracic activation versus abdominal repression. Moreover, genetic analysis revealed that both activities are Hox-dependent; Antp for activation and abdominal Hox factors for repression. To assess Hox factor binding to the DCRE, we performed comparative electromobility shift assays (EMSAs) using equimolar concentrations of Antp or Abd-A in the absence and presence of Exd/Hth. We found that Abd-A or Antp weakly bound the DCRE in the absence of Exd/Hth, whereas inclusion of Exd/Hth resulted in highly cooperative complex formation with either Hox factor (Fig 6A and 6B and 6D and 6E). However, the Abd-A complex bound DCRE more strongly than Antp, and Abd-A formed a third, slower migrating complex not seen with Antp (arrow in Fig 6E). Since previous studies had identified only two Hox sites, we scanned the DCRE and found a conserved region containing another potential Hox site preceded by a possible Exd site (TTATG, the ‘Hox0’ site and GAAT, the Exd0 site, see Fig 1A). Interestingly, this region coincides with the ‘BX0’ site that was footprinted by an abdominal Hox factor [26]. To assess the nature of the Abd-A and Antp Hox complexes on the DCRE, we assayed complex formation on a series of probes containing one or two linked Hox/cofactor binding sites (S4 Fig) as well as on DCRE probes containing point mutations in one, two, or all three Hox sites (S5 Fig). Neither Abd-A nor Antp formed strong complexes with Exd/Hth on probes containing individual Hox/cofactor sites. However, binding was increased cooperatively on probes containing two or more Hox/cofactor sites, and the number of molecular species observed increased according to the number of Hox/cofactor sites. These findings indicate that nearby Hox/cofactor binding sites contribute to cooperative DNA binding, even if the Hox/cofactor sites are suboptimal (the Exd0 sequence differs from the consensus sequence and the Exd1/Hox1 site contains an unfavorable nucleotide between the sites).

To assess the role of each Hox site in mediating DCRE-dependent repression and activation, we utilized site-selective mutagenesis in the *GD-lacZ* and *2XUD-lacZ* assays and quantified gene expression. Though the DCRE mediates both thoracic activation and abdominal repression in the context of the DMX, our assays effectively separate the two processes, allowing us to compare and quantify embryos as follows: 1) *GD-lacZ* assay: By stage 15 of embryogenesis no difference in β-gal levels was measured between cells across the thoracic segment (compare Slp2+ versus Slp2-negative thoracic cells in Fig 2F), indicating that localized DCRE-mediated thoracic activation is not observed at this stage of embryogenesis in the *GD-lacZ* assay. In addition, like the *GBE-lacZ*, no differences in levels were observed between Slp2-negative thoracic and abdominal cells in *GD-lacZ* embryos (see Fig 2F). Thus, thoracic DCRE-mediated activation was negligible in the *GD-lacZ* assay of stage 15 embryos, and we made direct comparisons between the T3 segment and the remaining thoracic and abdominal segments. 2) *UD-lacZ* assay: Our data indicates that the DCRE does not mediate significant abdominal repression in the *UD-lacZ* assay. In fact, quantification of β-gal intensity relative to Dll intensity in *2xUAS-lacZ* and *2xUD-lacZ* embryos reveals the DCRE significantly alters thoracic but not abdominal expression (Fig 3E). Thus, we normalized thoracic *2xUD-lacZ* β-gal levels to the A1 segment for each construct.

**Fig 7:**
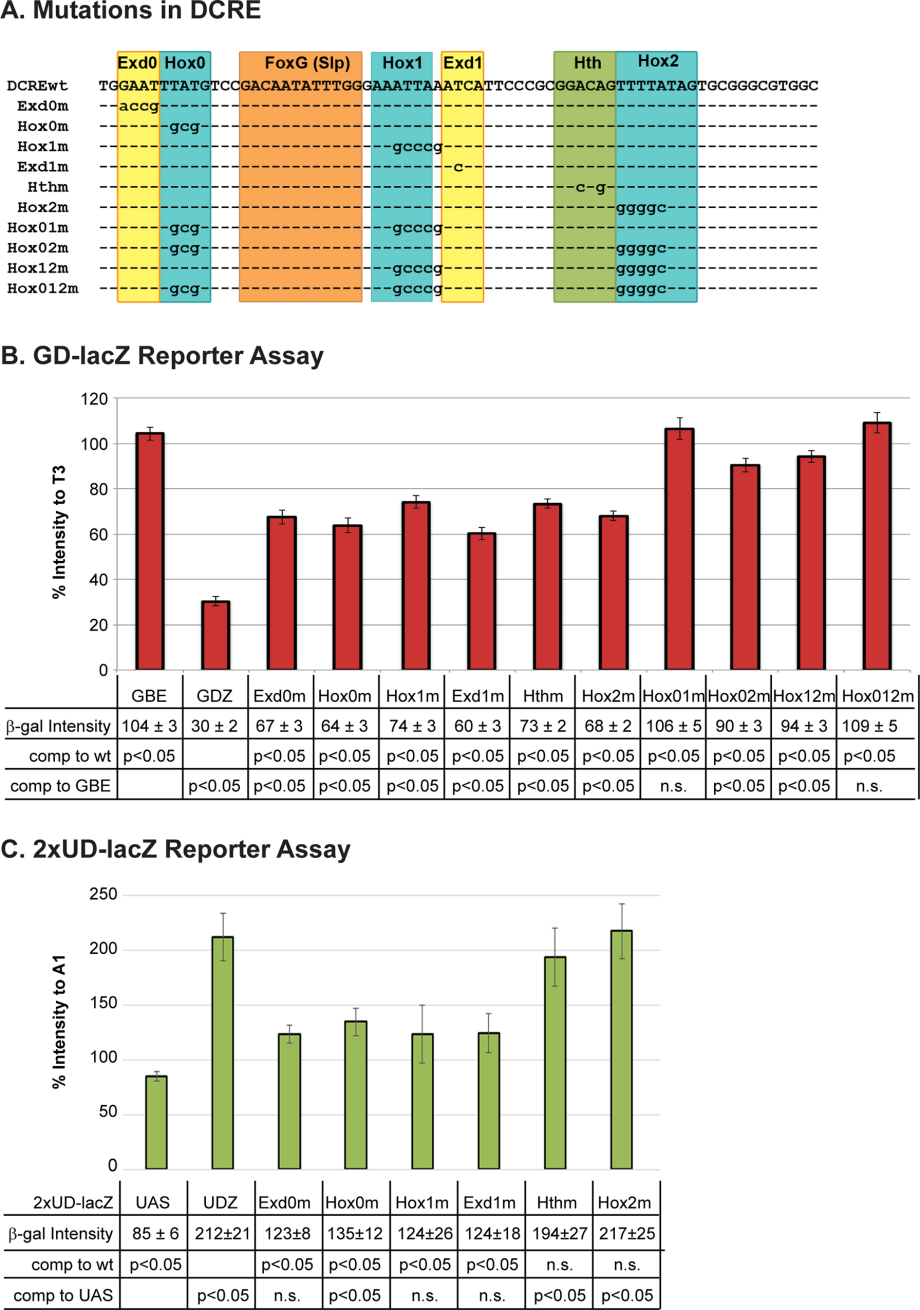
Hox-mediated repression and activation through the DCRE requires multiple binding sites. (A) Schematic of sequences used in the *GD-lacZ* and *2xUD-lacZ* reporter assays. DCREwt shown in first line, dashes indicate unchanged bases and changed bases are written out. (B) Quantification of abdominal intensity (relative to T3) of *GD-lacZ* wild-type and mutant reporters reveals that the DCRE is capable of partial repression unless the DCRE contains mutations in two or more Hox binding sites (Hox01M, Hox02M, Hox12M or Hox012M). (C) Quantification of thoracic intensity (relative to A1) of *2xUD-lacZ* wild-type and mutant reporters demonstrates that the Hth and Hox2 binding site are not required for thoracic activation in this assay. (Statistics shown in tables below charts, Welch’s t-test. n.s. = not significant).

To assess the dependence of DCRE abdominal repression on Hox/Hox cofactor sites, we first generated mutations in each Hox site or Hox cofactor site in the *GD-lacZ* assay. In each case, we found a significant decrease in DCRE-mediated repression in Slp+ abdominal cells indicating that all sites are required for optimal repression (Fig 7A and 7B and S6 Fig). However, no single point mutation abolished repression whereas double and triple Hox site mutations resulted in a complete loss of abdominal repression (Fig 7B and S6 Fig). These findings are consistent with previous mutation analysis on the DMX, which revealed double site mutations were required to yield full de-repression [28]. Taken together with the Hox DNA binding assays, these results indicate that the multiple linked Hox/cofactor sites in the DCRE can mediate robust Abd-A/Exd/Hth complex formation capable of abdominal transcriptional repression.

To assess whether thoracic activation by Antp/Exd/Hth complexes on the DCRE was also dependent upon the Hox binding sites, we analyzed the effect of single point mutations within each Hox site or Hox cofactor site using the *2XUD-lacZ* assay. We found that thoracic activation was dependent upon both the Hox0 and Hox1 and their associated cofactor sites (Exd0 and Exd1, respectively) but not the Hox2 or its associated Hth site (Fig 7C and S7 Fig). Hence, unlike abdominal repression, thoracic activation in the *2XUD-lacZ* assay is abolished by individual mutations in a subset of the Hox/cofactor binding sites.

### Evidence that a Hox TF collective model is consistent with DCRE regulation in the *Drosophila* embryo

**Fig 8:**
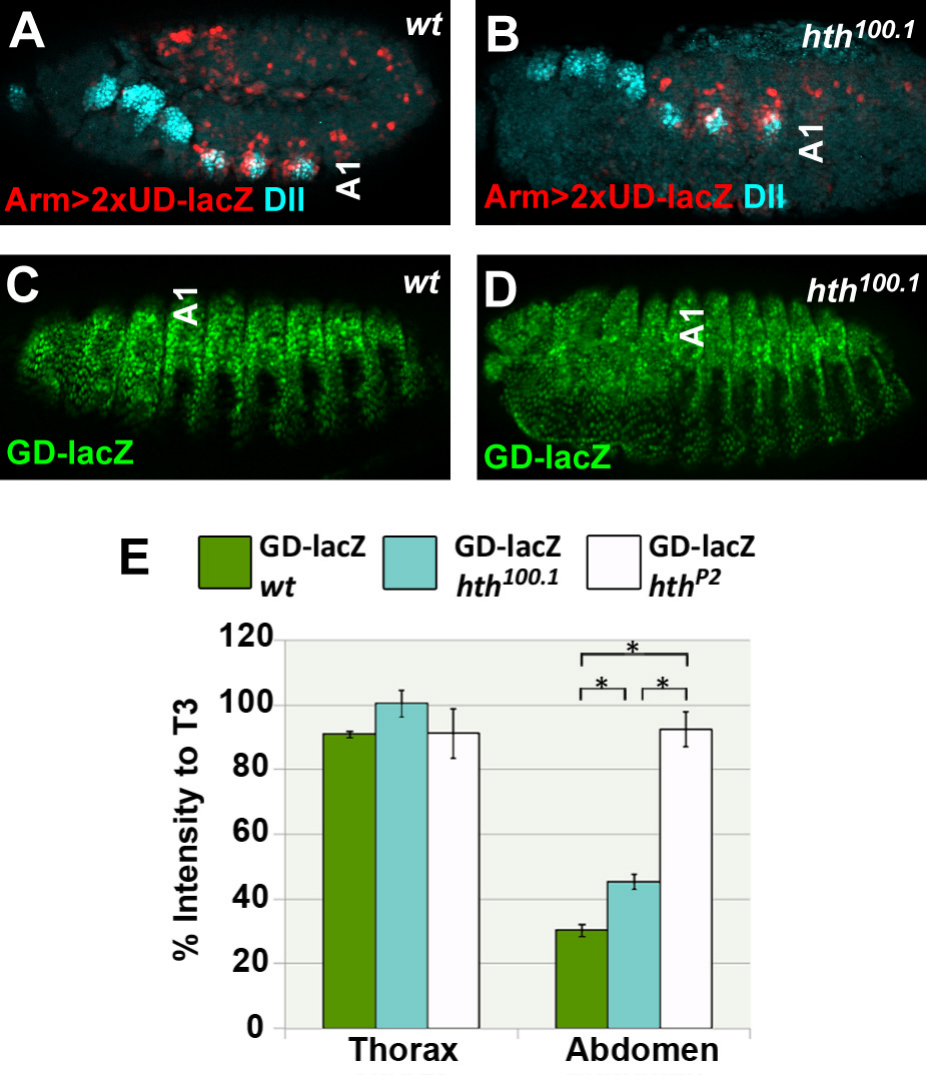
DCRE activity does not require the Hth homeodomain. (A, B) *ArmG4;2xUD-lacZ* reporter activity visualized in Stage 11 embryos immunostained with β-gal (red) and Dll (cyan) in either *wild type* (A) or *hth*^*100*.*1*^ (homeodomain-lacking) (B) embryos demonstrates similar levels of β-gal expression. (C, D) *GD-lacZ* reporter activity visualized in Stage 15 embryos immunostained with β-gal (green) in either *wild type* (C) or *hth*^*100*.*1*^ embryos (D). (E) Quantification of β-gal intensity relative to T3 of *GD-lacZ* in *wild-type*, *hth*^*100*.*1*^, and *hth*^*p2*^ (strong hypomorph) embryos demonstrates that *GD-lacZ* repression requires Hth, but not the Hth homeodomain.

Of the three major models of CRM function (billboard, enhanceosome, TF collective), our results are most congruent with Hox factors, especially Abd-A, functioning as a TF collective with Exd and Hth on the DCRE. First, unlike the all or none activity predicted by the enhanceosome model, the DCRE mediates significant repression even when individual TF binding sites are mutated in both the *GD-lacZ* and DMX assays (Fig 7B and S6 Fig and [28]). Second, we found that unlike the independent binding of TFs predicted by the billboard model, Abd-A/Exd/Hth forms multiple cooperative complexes using several distinct binding sites, and can even do so with individual binding sites mutated (S4 Fig and S5 Fig). An additional postulate of the TF collective is that not all TFs of the collective are required to directly bind DNA to contribute to transcriptional activity. Indeed, while individual point mutations within the sole Hth binding site decreased DCRE-mediated abdominal repression in the *GD-lacZ* assay, significant repression was still observed in this assay as well as in the context of the full DMX (Fig 7 and [28]). As a further test of this idea, we used a *hth* point mutation (allele *hth*^*100*.*1*^) that inserts a premature stop codon to generate homeodomain-less Hth proteins [43]. Importantly, this allele mimics a naturally occurring alternative splice isoform of Hth (as well as the vertebrate Meis proteins), and while these Hth∆HD proteins fail to directly bind DNA, they still interact with and translocate Exd into the nucleus [44]. As expected, we found that *2XUD-lacZ* activated thoracic expression in *ArmG4;hth*^*100*.*1*^ embryos to a level similar to wild type embryos, demonstrating that Hth DNA binding is not required for this activity (Fig 8A and 8B). We also analyzed *GDZ* activity in *hth*^*100*.*1*^ embryos, and found significant repression in abdominal Slp2+ cells, albeit, the level of repression was reduced in *hth*^*100*.*1*^ embryos compared to *wild type* embryos (45% versus 70% repression, Fig 8C–8E). By comparison, repression is abolished in *hth*^*P2*^ null embryos (Figs 8C and 4C). This data is consistent with a previous study that reported normal *Dll* and *DMX* expression in *hth*^*100*.*1*^ embryos [44]. We confirmed this finding by quantifying *DMX-lacZ* expression in *wild type* and *hth*^*100*.*1*^ embryos and found no significant difference in abdominal repression (S8 Fig). We also tested Hox point mutant-carrying *GD-lacZ* reporters in the context of *hth*^*100*.*1*^ embryos. As expected, point mutations within the Hox2 site, which is linked to the adjacent Hth site, did not further decrease *GD-lacZ* dependent repression in *hth*^*100*.*1*^ embryos (S6 Fig). In contrast, Hox1 point mutations in this genetic background lost all repression activity, a result that is consistent with the fact that multiple Hox/cofactor sites need to be mutated to abolish DCRE-mediate repression (Fig 7B and S6 Fig).

Next, we assessed whether the homeodomain-less Hth protein can contribute to cooperative Abd-A DNA binding on the DCRE. We also tested the role of Exd DNA binding on complex formation using an Exd protein containing a point homeodomain mutation (N51A) that disrupts DNA binding. Importantly, purified Exd/Hth∆HD (Fig 6F) and Exd51A/Hth (Fig 6G) heterodimers did not significantly bind the DCRE in the absence of Abd-A, even when added at a concentration three times higher than the wild type heterodimer (compare second column of each EMSA to wild type Exd/Hth binding in Fig 6E). Inclusion of Abd-A, however, revealed that either DNA binding deficient heterodimer (Exd/Hth∆HD or Exd51A/Hth) stimulated significant cooperative Hox complex formation on the DCRE (Fig 6F and 6G). To determine the independent role of Hth and Exd protein in complex formation, we performed EMSAs using Abd-A with only purified Exd or Hth (Fig 6H and 6I). In contrast to the DNA binding deficient heterodimers, the addition of equimolar concentrations of Exd or Hth alone with Abd-A did not yield significant complex formation on the DCRE (Fig 6H and 6I). These findings are consistent with the TF collective model of CRM function in which protein-protein interactions between Exd and Hth contribute to cooperative TF complex formation with Abd-A on the DCRE.

**Fig 9:**
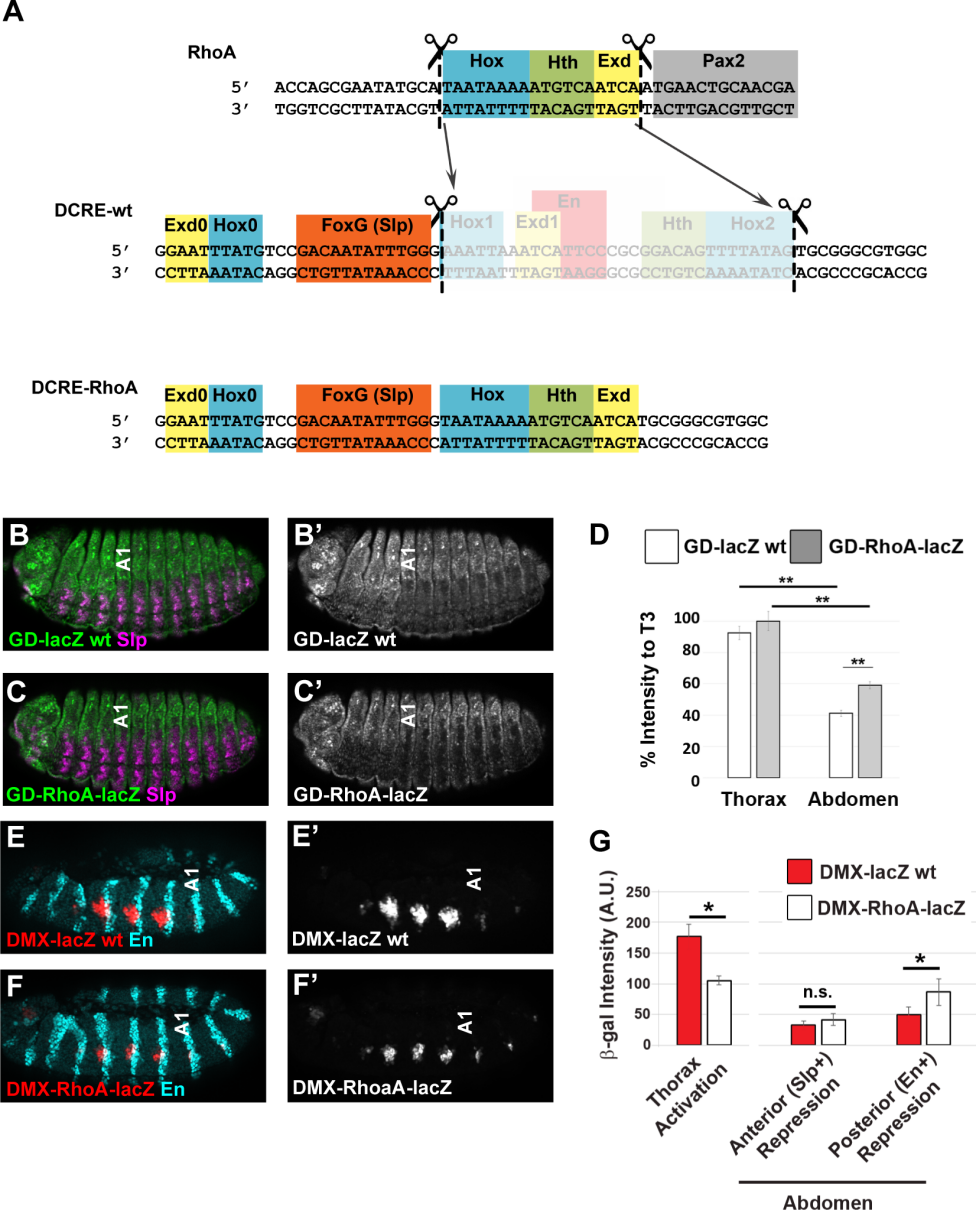
The DCRE recruits a Hox TF collective. (A) Sequences of DCRE, RhoA, and DCRE-RhoA with Hox, Hth, Exd, FoxG, En, and Pax2 binding sites highlighted. Note that DCRE-RhoA lacks the En binding site required for posterior compartment repression. (B-C) *GD-lacZ* wild-type (B) and *GD-RhoA-lacZ* (C) activity visualized in Stage 15 embryos immunostained with β-gal (green or white) and Slp (magenta) demonstrates that the DCRE-RhoA element is capable of substantial abdominal repression. (D) Quantification of β-gal intensity relative to T3 segment shows that the DCRE-RhoA element is capable of substantial repression in the abdomen relative to the thorax, though it does not repress to DCRE wild-type levels. (** = p<0.01, Welch’s t-test). (E-F) Visualization of *DMX-lacZ* wild-type (E) and *DMX-RhoA-lacZ* (F) activity in stage 11 embryos immunostained for β-gal (red) and En (cyan) reveals that the DCRE-RhoA element represses in En negative cells of the abdomen. Note, that the thoracic levels activated by *DCRE-RhoA-lacZ* are decreased relative to *DMX-lacZ* wild-type. (G) Quantification of β-gal intensity in the thorax, the anterior abdominal compartment, and the posterior abdominal compartment of *DMX-lacZ* and *DMX-RhoA-lacZ* embryos. (* p<0.01, Tukey’s Test).

To determine if different configurations of Hox/Exd/Hth sites could confer similar transcriptional outcomes, we replaced a subset of the Hox/cofactor sites within the DCRE with a distinct set of sites from another Hox-regulated CRM. Previous studies revealed that a *rhomboid* CRM (RhoBAD) mediates transcriptional activation in sensory organ precursors by integrating an Abd-A/Hth/Exd complex with the Pax2 TF [45,46]. The RhoBAD CRM contains separable binding sites for Pax2 and Abd-A/Hth/Exd (Fig 9A). To determine if the Hox/Hth/Exd sites found in RhoBAD can function in transcriptional repression in the DCRE, we replaced the Hox1/Exd1-Hox2/Hth sites of the DCRE with the Hox/Hth/Exd sites from RhoBAD (*DCRE-RhoA*, Fig 9A). This fusion transgene lacks the RhoBAD Pax2 site necessary for activation but contains the DCRE FoxG (Slp) sites as well as the Exd0/Hox0 sites that contribute to, but are not sufficient, for mediating repression. We found that the *GD-RhoA-lacZ* was able to substantially repress gene expression in Slp+ abdominal cells, although not as strongly as the wild type DCRE (Fig 9B–9D). To determine if this modified element was sufficient to repress the DMX enhancer in the abdomen, we compared the activity of *DMX-lacZ* and *DMX-RhoA-lacZ* transgenes. Since the DCRE-RhoA element lacks the En site required for posterior compartment repression, significant de-repression in En+ cells was expected and observed in *DMX-RhoA-lacZ* (Fig 9F). In contrast, repression of the *DMX-RhoA-lacZ* was comparable to that of *DMX-lacZ* in Slp+ abdominal cells (Fig 9E–9G). However, similar to *DMEact-lacZ*, the *DMX-RhoA-lacZ* configuration of sites expressed decreased levels of β-gal in the thorax compared to the wild type *DMX-lacZ* (Fig 9E–9G). Altogether, these findings demonstrate that while the *DMX-RhoA* configuration of Exd/Hth/Hox sites can mediate significant abdominal repression in Slp+ cells, this configuration of sites failed to perform two other DCRE-dependent activities (posterior compartment repression in the abdomen and conditional activation in the thorax).

## Discussion

While it has been established that CRMs regulate a gene’s spatial and temporal transcription expression pattern, we are only now appreciating the complexity of CRMs regarding the number of inputs required to yield cell/tissue specific patterns. In this study, we built upon our knowledge of how the DCRE CRM integrates Hox, Exd, and Hth TFs to ensure precise *Dll* expression during leg specification. Using quantifiable transgenic reporter and DNA binding assays, we found that the DCRE can recruit either Hox-based repression (Abd-A/Ubx) or activation (Antp) complexes using multiple Hox/Exd and/or Hox/Hth sites. Importantly, the DCRE Hox, Exd, and Hth binding sites and flanking regions are highly conserved across *Drosophilid* species, yet our studies reveal that an abdominal Hox TF collective can mediate robust cell-specific (Slp+) repression through flexible combinations of Hox/co-factor binding sites. However, the DCRE regulates at least two additional cell-specific transcriptional outcomes, suggesting that the DCRE CRM TF binding sites are under added constraints and maintains high sequence conservation to mediate multiple cell-specific outputs. Thus, our findings provide new insights into Hox specificity, CRM function, and CRM conservation.

### Hox specificity: Integrating multiple Hox-cofactor sites

In spite thirty years of study, we lack a general understanding of how Hox factors gain sufficient specificity to differentially regulate cell fates along the anterior-posterior axis of metazoans. As monomers, Hox factors bind highly similar DNA sequences *in vitro* [47,48]. The discovery of two general Hox cofactors that also encode TFs, Exd (vertebrate Pbx) and Hth (vertebrate Meis), suggested that the formation of TF complexes enhances Hox DNA binding affinity and specificity [32,49,50]. Consistent with this idea, the biochemical characterization of Exd/Hox binding sequences using SELEX-seq revealed DNA binding preferences between Hox factors are enhanced by Exd (termed latent specificity) [51]. The Forkhead (Fkh) CRM, for example, contains a unique Hox/Exd site that is specifically bound and regulated by a Sex combs reduced (Scr)/Exd complex [52,53]. More recent studies revealed that Exd also enhances Hox specificity by binding several low affinity sites. Crocker et al. found two CRMs from the *shavenbaby* (*svb*) locus that are activated in the abdomen by either Ubx/Exd or Abd-A/Exd complexes via low affinity sites [54]. Altering these sequences to high affinity Hox/Exd sites resulted in a loss of Hox specificity and transcriptional activation by anterior Hox factors. These findings suggest high affinity Hox/Exd sites are more likely to be pan-Hox target sequences regulated by numerous Hox factors whereas low affinity Hox/Exd sites provide specificity.

**Fig 10:**
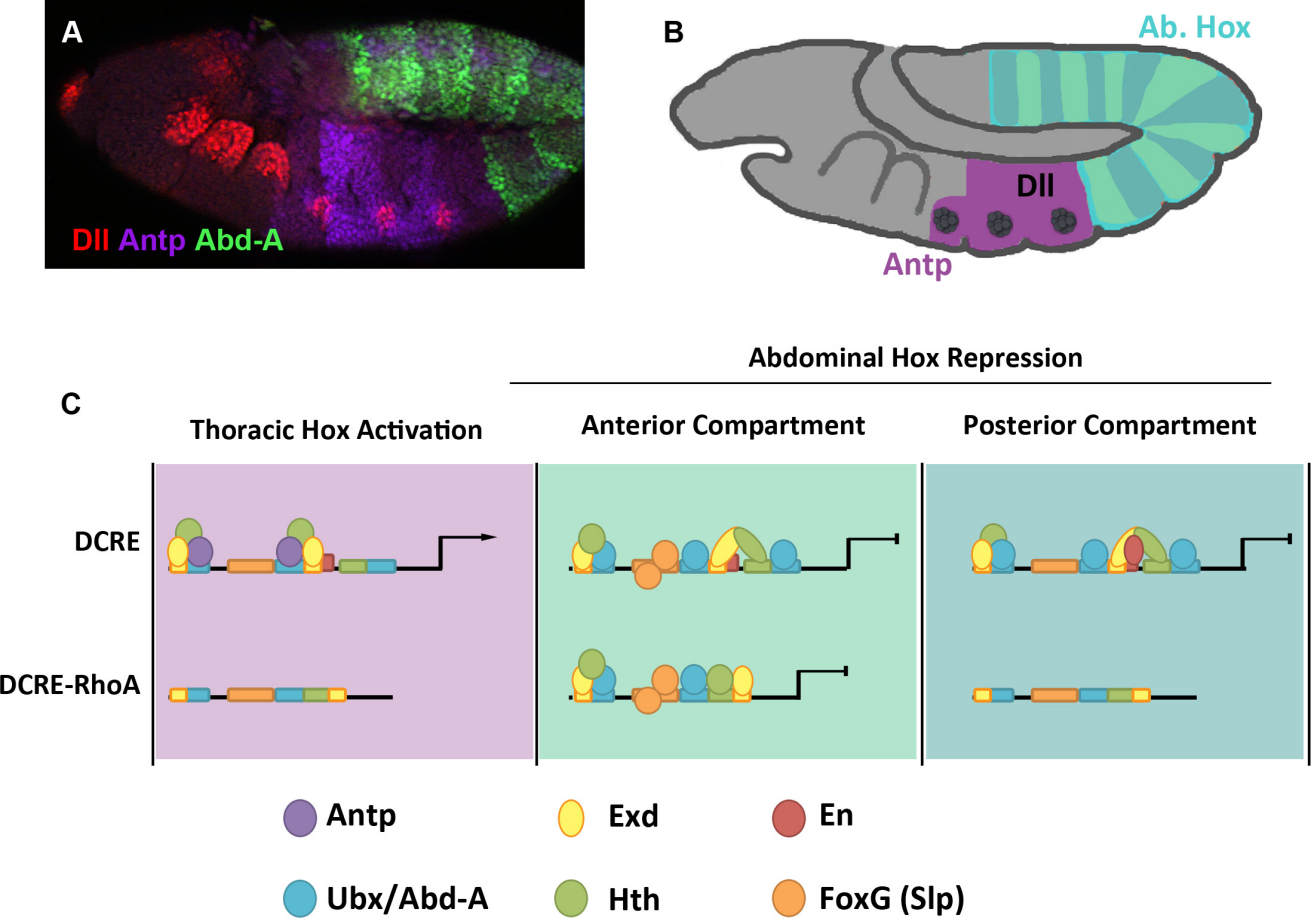
Model of Hox repression and activation via the DCRE. (A) Stage 11 embryo immunostained for Dll (red), Antp (magenta), and Abd-A (green). (B) A model of a stage 11 embryo with thoracic Dll region noted in grey, the thoracic Hox factor, Antp in purple, and the abdominal Hox factors, Ubx and Abd-A in blue. (C) The three functions of the DCRE are noted and background color corresponds to model embryo in panel B. Top row: In the thorax, the DCRE is bound by a complex of Antp, Hth, and Exd on two of the Hox/cofactor sites. In the anterior compartment of the abdomen, DCRE is bound by the abdominal Hox factors, Abd-A or Ubx, along with Hth and Exd on all three Hox/cofactor sites as well as the repression cofactor, Slp. In the posterior compartment of the abdomen, DCRE is bound by the abdominal Hox factors, Abd-A or Ubx, along with Hth and Exd on all three Hox/cofactor sites as well as the repression cofactor, En. Bottom Row: A new configuration of Hox sites in the DCRE, the DCRE-RhoA fusion, is only capable of performing one of the three DCRE functions, repression in the anterior abdominal compartment.

In this study, we show that the DCRE mediates two opposing transcriptional outcomes using three linked Hox-cofactor binding sites. In the thorax, an Antp/Exd/Hth complex activates largely via two Hox/Exd sites, whereas the linked Hox/Hth sites are less important for DCRE-mediated activation. In the abdomen, all three Hox sites contribute to repression via the recruitment of several Abd-A/Exd/Hth complexes. Hence, the most specific Hox site within the DCRE is the linked Hth/Hox site that mainly contributes to abdominal repression by binding Abd-A and Ubx (Fig 10). In fact, directly linked Hth/Hox sites may be preferentially regulated by posterior Hox factors as the Abd-A specific target gene *rhomboid* (*rho*) contains a CRM that is activated via a linked Hth/Hox site [45,46]Additionally, biochemical studies using vertebrate Hox factors revealed that only posterior Hox factors form direct complexes with the Meis factor on DNA [55]. In contrast, both Exd/Hox sites within the DCRE are regulated by both thoracic Hox factors (activation) and abdominal Hox factors (repression) (Fig 10). Sequence analysis reveals that neither DCRE Exd/Hox site is optimal as an extra nucleotide is inserted between the Hox1 and Exd1 site whereas the Exd0 site has several mismatches to its consensus sequence (S1 Fig). Moreover, DNA probes containing isolated Exd/Hox sites from the DCRE are poorly bound by Hox/Exd proteins, whereas combining these suboptimal sites resulted in the formation of Hox complexes that contribute to gene regulation. Thus, the DCRE uses multiple Hox/Hox cofactor sites to recruit distinct complexes that mediate two opposing transcriptional outcomes along the anterior-posterior axis.

While a repression function for the DCRE was expected based on previous studies, the DCRE also contributes to Hox-mediated activation in the thorax. We termed the DCRE a ‘conditional’ activator in the thorax because it fails to initiate transcription, but when coupled to a ubiquitous activation element the DCRE enhances transcription in a subset of thoracic cells. Importantly, the cells that activate the DCRE derive from the endogenous *Dll* expression domain, and the DCRE contributes to activation of the DMX leg enhancer in an *Antp*-dependent manner. These data support the model that Antp and Exd/Hth are required for the conditional activation function of the DCRE. However, it is currently unclear why this activity is restricted to the Dll+ leg/wing primordium since Antp and Exd/Hth are broadly expressed throughout the thorax. One possibility is that, much like in the abdomen, an additional factor(s) interacts with the DCRE to provide position-specificity.

### Evidence for a Hox TF collective model of CRM function

How CRMs integrate transcription factor complexes to mediate cell-specific outputs remains an active area of study. The two best-known CRM models are the enhanceosome and the billboard. These models can be seen as extreme opposite ends of the spectrum of rigidity and constraints (enhanceosome) versus flexibility and adaptability (billboard), with most CRMs likely to contain aspects of both models. Since many TFs use protein-protein interactions to promote cooperative complex formation on DNA, these interactions often place constraints on the order, orientation, and spacing of TF binding sites within CRMs. Hence, cooperative DNA binding has often been seen as evidence consistent with an enhanceosome model of CRM function. Dimerization between TFs such as the basic Helix-Loop-Helix (bHLH) proteins and retinoic acid receptors, for example, results in the formation of TF complexes that bind palindromic sequences with restrictions on distances between individual binding sites. In 2012, Junion et al proposed an alternative role for protein-protein interactions between TFs [22]. Using a series of chromatin immunoprecipitation experiments, the Furlong lab found that a group of five TFs regulate a set of cardiac CRMs in the *Drosophila* embryo. Sequence analysis of co-regulated CRMs revealed combinatorial binding of these TFs does not require specific motif organization, a finding that is also consistent with the billboard model of CRM function. However, unlike the billboard, the TF collective does not require individual DNA binding sites for every TF to mediate appropriate functional outputs. Instead, a TF collective uses a combination of clustered DNA binding sites and protein-protein interactions to recruit large-scale TF complexes containing all the members of the collective. Although the biochemical basis of TF interactions between the five TFs was not explored, previous studies did find that a subset of these TFs form direct protein-protein interactions. Thus, Junion et al proposed the TF collective model of CRM function that predicts a common group of TFs can form many different cooperative complexes via multiple interactions between TFs, which results in greater CRM flexibility rather than rigidity in DNA binding site organization [22].

In this study, we provide evidence consistent with a Hox TF collective regulating early *Dll* expression in the *Drosophila* embryo. First, we show that the DCRE uses at least three distinct Hox sites that are each linked to an adjacent Exd or Hth binding site to recruit functional Hox complexes. Focusing on abdominal Hox-mediated repression, we used DNA binding assays and a synthetic reporter system (*GD-lacZ*) to reveal the following correlations between DNA binding affinity and transcriptional repression: 1) The wild type DCRE containing all three Hox sites yielded the strongest Abd-A/Exd/Hth binding and transcriptional repression in abdominal Slp+ cells. 2) Individual point mutations within any one Hox site partially compromised complex formation and repression. However, significant repression was still observed in the *GD-lacZ* assay, and in the *DMX-lacZ* assay single point mutations were still able to mediate abdominal repression in Slp+ cells in the DMX reporter [28]. 3) Mutations that compromise any two Hox sites or two Hox co-factor sites further decreased Abd-A/Exd/Hth complex formation, and abolished *GD-lacZ*-mediated abdominal repression. Consistent with the TF collective model, we found that Abd-A could still form robust complex formation on the DCRE even in the presence of DNA binding deficient Exd or Hth proteins, and genetic studies revealed that the DNA binding activity of one of the factors (Hth∆HD) is not required to mediate significant abdominal repression or thoracic activation. Moreover, we replaced the Hox1/Exd1-Hth/Hox2 sites with a distinct configuration of Exd/Hth/Hox sites from a different Abd-A regulated CRM and observed significant repression in both the *GD-lacZ* and *DMX-lacZ* assays (Fig 10). In total, these data demonstrate that, in the anterior compartment of the abdomen, multiple Hox/Exd/Hth binding site configurations can recruit a Hox TF collective capable of mediating robust transcriptional outputs.

Interestingly, other Hox CRMs also contain characteristics consistent with TF collective enhancers. For example, congruent with variable binding of TFs in a collective, comparison of five mouse hindbrain enhancers controlled by HoxA1 and HoxB1 along with the Exd/Hth homologs, Pbx and Meis demonstrated that the presence, orientation, location, and sequence of the Meis sites are highly variable [56–61]. Additionally, the Hth homeodomainless protein is functional on other Hox-regulated CRMs, including the Fkh250 and Lab550 CRMs in *Drosophila* embryos [44]. Together, these results suggest that the DCRE is not unique among Hox CRMs in fitting the TF collective model.

### The DCRE is a pleiotropic CRM: A proposed role for multiple cell-specific outputs on CRM sequence conservation

An unanswered question emerges from these studies: if interactions between members of the Hox TF collective permit added flexibility in binding site configurations, why is the DCRE so highly conserved across *Drosophilid* species? One possible reason is that the DCRE mediates multiple opposing Hox-dependent outputs, which places added constraints on sequence conservation. For example, while replacing the Hox1/Exd1-Hth/Hox2 sites with the Exd/Hth/Hox configuration from the RhoBAD CRM can mediate strong repression in Slp+ anterior compartment cells, this configuration fails to repress gene expression in the posterior compartment due to the lack of an En binding site. Similarly, DCRE reporters containing this configuration of Hox/Hox cofactor sites also yielded lower levels of β-gal expression in the thorax, consistent with the idea that Antp fails to regulate linked Hth/Hox sites. Hence, we propose that the dual repression mechanisms of the DCRE in the anterior and posterior compartments of the abdomen as well as its conditional activation function in the thorax requires numerous TF sites, which thereby places evolutionary pressure to maintain sequence conservation.

Several different hypotheses have been proposed for why some CRMs are highly conserved, including pleiotropic functions of CRMs placing added constraints on conservation [62–65]. Moreover, a recent vertebrate study comparing TF binding to syntenic regions of mouse and human genomes revealed that the most highly conserved TF binding activities were found on CRMs with pleiotropic functions in multiple cell types [66]. This study also noted that pleiotropic CRMs enrich for the co-association of many TFs. While this study did not score each CRM for nucleotide identity, their findings are consistent with our functional study on the DCRE and suggest that pleiotropy places added constraints on CRM sequence conservation.

## Materials and Methods

### Generation of transgenic fly lines

The DMX [28], DMEact (basepairs 1–661 of DMX), 3xGrainyHead binding element1 (3xGBE) [67], and 2xUAS elements were generated by PCR (sequences available upon request). DCRE-containing plasmids were created by ligating annealed complementary oligonucleotides containing restriction enzyme overhangs into the 3xGBE, or 2xUAS plasmids. Sequences of DCRE mutants are located in the figures. All enhancers were subcloned into the placZAttB plasmid. UAS-Abd-A was generated by PCR and subcloned into the pUAST-AttB plasmid. All plasmids were confirmed by DNA sequencing. Transgenic fly lines were generated by ΦC31 integration into the 51C insertion site [68] (Injections by Rainbow Transgenics).

### Drosophila stocks and immunostaining

The following fly lines were used: *Antp*^*25*^, *Ubx*^*1*^, *hth*^*P2*^, *PrdG4*, *ArmG4* (Bloomington Stock Center); *Abd-A*^*MX1*^, *Ubx*^*Mx12*^*Abd-A*^*M1*^, *Slp*^*∆34b*^, *UAS-Ubx* (Richard Mann, Columbia University, NY, USA); *hth*^*100*.*1*^ (Kurant et al., 2001); *UAS-Abd-A* (this work). Embryos were collected, fixed and stained using standard procedures at 25°C except for *PrdG4;UAS-Abd-A* and *PrdG4*:*UAS-Ubx* experiments which were performed at 18°C to lower Gal4 activity. The following primary antibodies were used: En (mouse 1:10) (Developmental Studies Hybridoma Bank, DSHB), Antp (mouse 1:50) (DSHB), Abd-A (guinea pig 1:500) (Li-Kroeger et al., 2008), Ubx (mouse 1:20) (Richard Mann); Vestigial (rabbit 1:25) (Sean Carroll, University of Wisconsin-Madison, WI, USA); and β-gal (chicken 1:1000) (Abcam). Antibodies were generated against Slp2 (amino acids 1–275) and Dll (full-length) using purified His-tagged proteins injected into rats (Cocalico Biologicals). Both the Slp2 and Dll sera were used at 1:500. All immunostains were detected using fluorescent secondary antibodies (Jackson Immunoresearch Inc. or Alexa Fluor, Molecular Probes). For quantitative analysis of gene expression, sets of embryos were harvested, fixed, and imaged under identical conditions at the same time. When possible, age-matched siblings were analyzed. For *GD-lacZ* and *UD-lacZ* assays, images used for quantification were taken using a single exposure time and normalized to segment T3, A1, or Dll expression levels within the same embryo as indicated. Pixel intensities and areas were measured using NIH-ImageJ software.

### Protein purification and EMSAs

The following proteins were purified from BL21 cells as previously described [27]: His-tagged Abd-A [69]; Antp [27]; his-Hth [70] and untagged Exd heterodimers [27]; his-Hth∆HD/Exd heterodimers [51]; his-Exd51A/Hth heterodimers [56]; his-Hth and his-Exd. Purified proteins were confirmed using SDS-PAGE and Coomassie blue staining and concentrations measured by Bradford assay. EMSAs were performed as previously described using native polyacrylimide gel electrophoresis [56]. Probes were used at 0.36 μM, and protein concentrations are noted in figure legends. The dried acrylamide gels were exposed to a phosphor screen for imaging using a StormScanner (GE Healthcare). Densitometry was performed using ImageQuant 5.1 software. All EMSA experiments were performed in triplicate.

## Supporting Information

S1 Fig Conservation of the Dll leg enhancer. (A) Comparison of the four versions of the DCRE element, bps relative to the Dll304 enhancer (bp 1–877). (B) Conservation plot of the Dll304 enhancer (base pairs 1–877) generated with the UCSC Genome Browser (http://genome.ucsc.edu/, [71]). Black box indicates the DCRE element as defined by breaks in sequence conservation. Blue box indicates the original region identified as the BX-NRE element. Below, sequence alignment of the DCRE. *Drosophila melanogaster* sequence is listed, conserved base-pairs are marked as dashed lines, and non-conserved base-pairs are noted in each species. The known repression binding sites are highlighted. (C) Comparison of consensus binding sites for Pbx/Hox, Meis/Hox, and Fkh to the binding sites found in the DCRE. Base-pairs that differ from consensus are marked in red.
(TIF)

S2 Fig The 2xUD-lacZ reporter is not active without a Gal4 driver. (A-B) Neither the *2xUAS-lacZ* reporter (A) nor the *2xUD-lacZ* reporter (B) express β-gal without a Gal4 driver. All panels are lateral views of Stage 11 embryos immunostained for β-gal (white).
(TIF)

S3 Fig Quantification of GD-lacZ expression in Hox mutant embryos. (A). *wild-type* GD-lacZ embryo. (B) *GD-lacZ*; *abd-A*^*M1*^ embryo shows that the Ubx Hox factor compensates for loss of Abd-A. (C) A *GD-lacZ*; *Ubx*^*MX1*^ shows that Abd-A compensates for Ubx in segments A2-A7 where it is expressed. (D) A *GD-lacZ*; *abd-A*^*M1*^, *Ubx*^*Mx12*^ embryo indicates that the abdominal Hox factors are required for DCRE-mediated repression in the *GD-lacZ* assay. (E) Quantification of β-gal intensity of panels A-D relative to T3. All images are lateral views of Stage 15 *GD-lacZ* embryos immunostained for β-gal (white).
(TIF)

S4 Fig Contribution of linked Hox-Exd and Hox-Hth sites. (A) Schematic of the DCRE probes used for EMSAs with Hox, Exd, and Hth sites highlighted. Percent probe bound ± S.E.M. by Hox/Exd/Hth is listed at right for each probe. (B) EMSAs using 25 nM Exd/Hth (E/H, yellow boxes) and 77.5 nM Abd-A (AA blue boxes) on probes containing one or two Hox/cofactor paired sites as listed. (C) EMSAs using 25 nM Exd/Hth and 310 nM Antp (An, magenta boxes) as shown on probes containing one or two Hox/cofactor paired sites. Note that both Abd-A and Antp form complexes on paired Hox/cofactor sites, but Abd-A binds more strongly than Antp.
(TIF)

S5 Fig Effect of Hox site mutation on binding of AbdA/Exd/Hth complexes to the DCRE. (A) Schematic of the DCRE probes used for EMSAs with Hox, Exd, and Hth sites highlighted. (B) EMSAs performed on the DCRE full-length probes as labeled below each gel. Titration of AbdA protein (concentrations from 37.5 nM to 300 nM) on the DCRE probes as labeled with 25 nM purified Exd/Hth dimer.
(TIF)

S6 Fig DCRE mediated repression in Hox/cofactor mutant reporters. (A-N) Visualization of GD-lacZ reporter in *wild-type* (*wt*) (A-L) or *hth*^*100*.*1*^ (M-N) embryos. For DCRE sequences, see S5 Fig. All images are lateral views of Stage 15 embryos immunostained for β-gal (green). Each GD-lacZ reporter variant is labeled in green in the lower left hand corner of each embryo image, and the genotype of the embryos are labeled in the upper right hand corner.
(TIF)

S7 Fig DCRE mediated activation in Hox/cofactor mutant reporters. (A-H) Visualization of 2xUD-lacZ reporters in *wild-type* embryos. For DCRE sequences, see S5 Fig. All images are lateral views of Stage 11 embryos, segments T2, T3, and A1 shown, immunostained for β-gal (red) and AbdA (cyan). Each 2xUD-lacZ reporter variant is labeled below each embryo.
(TIF)

S8 Fig Robust abdominal repression does not require the Hth homeodomain. (A-B) Stage 11 DMX-lacZ embryos immunostained for β gal (red) and Dll (cyan), in either a wild type (A) or *hth*^*100*.*1*^ mutant (B) background demonstrate that Dll and DMX-repression remain normal in absence of the Hth homeodomain. (C) Quantification of DMX-lacZ in wild type and *hth*^*100*.*1*^ embryos reveals no significant difference in β gal levels of the abdomen or thorax.
(TIF)
