# Supplementary figures and images for: A Hox Transcription Factor Collective Binds a Highly Conserved Distal-less cis-Regulatory Module to Generate Robust Transcriptional Outcomes
Source: PLoS Genet. 2016 Apr 8;12(4):e1005981. doi: 10.1371/journal.pgen.1005981 (PMC4825978; doi:10.1371/journal.pgen.1005981)

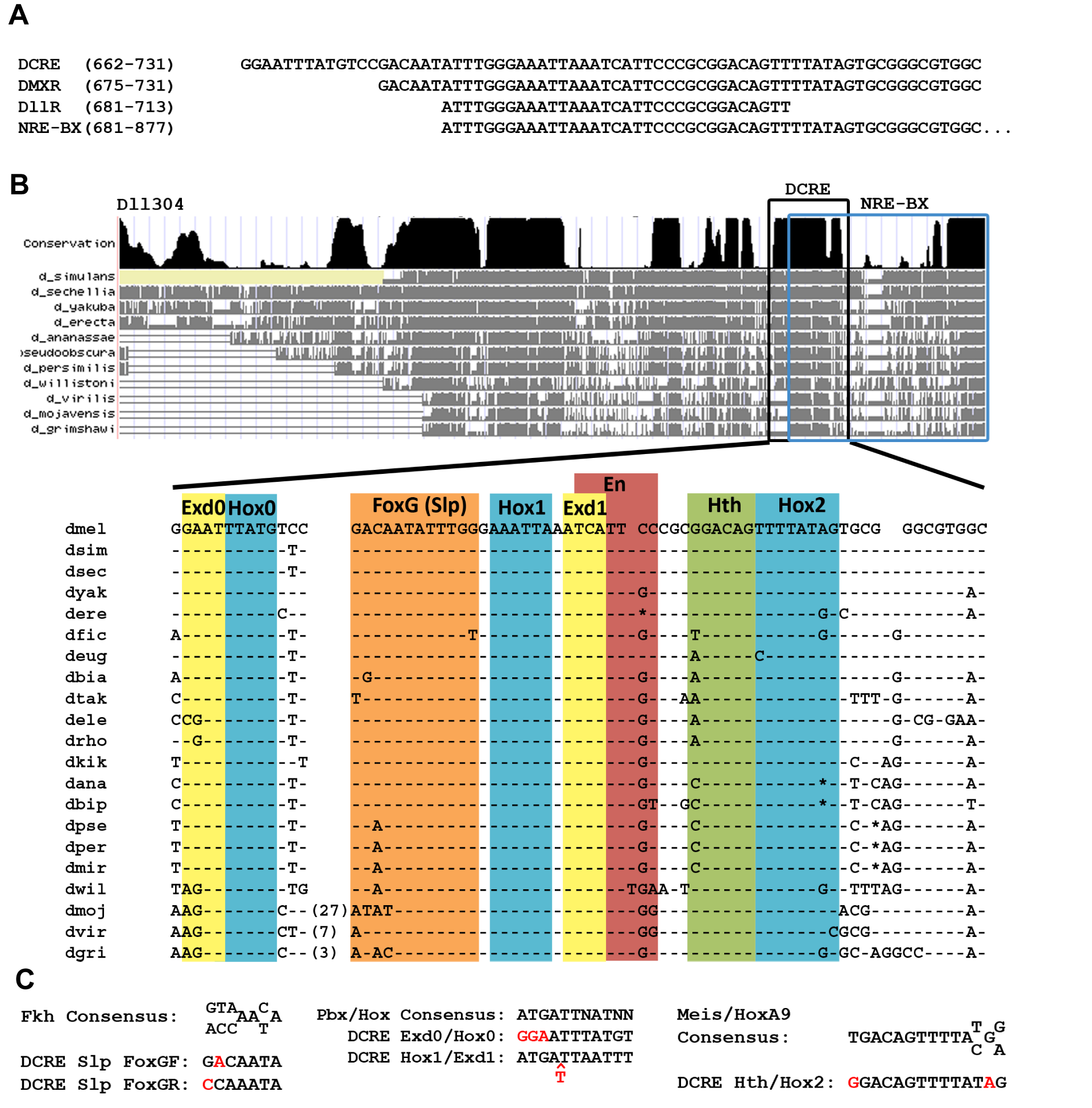

Supplement: S1 Fig — (A) Comparison of the four versions of the DCRE element, bps relative to the Dll304 enhancer (bp 1–877). (B) Conservation plot of the Dll304 enhancer (base pairs 1–877) generated with the UCSC Genome Browser (http://genome.ucsc.edu/, [71]). Black box indicates the DCRE element as defined by breaks in sequence conservation. Blue box indicates the original region identified as the BX-NRE element. Below, sequence alignment of the DCRE. Drosophila melanogaster sequence is listed, conserved base-pairs are marked as dashed lines, and non-conserved base-pairs are noted in each species. The known repression binding sites are highlighted. (C) Comparison of consensus binding sites for Pbx/Hox, Meis/Hox, and Fkh to the binding sites found in the DCRE. Base-pairs that differ from consensus are marked in red. (TIF) [file pgen.1005981.s001.tif]

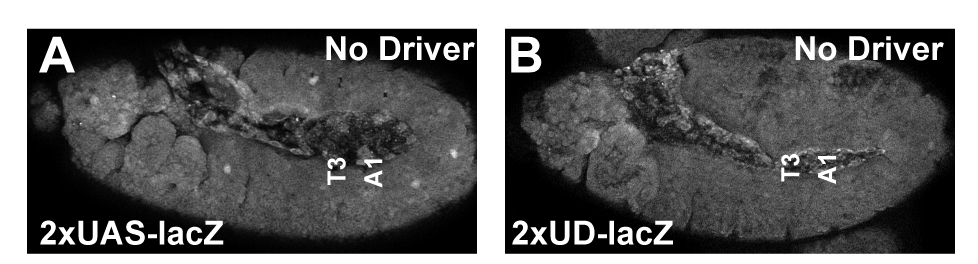

Supplement: S2 Fig — (A-B) Neither the 2xUAS-lacZ reporter (A) nor the 2xUD-lacZ reporter (B) express β-gal without a Gal4 driver. All panels are lateral views of Stage 11 embryos immunostained for β-gal (white). (TIF) [file pgen.1005981.s002.tif]

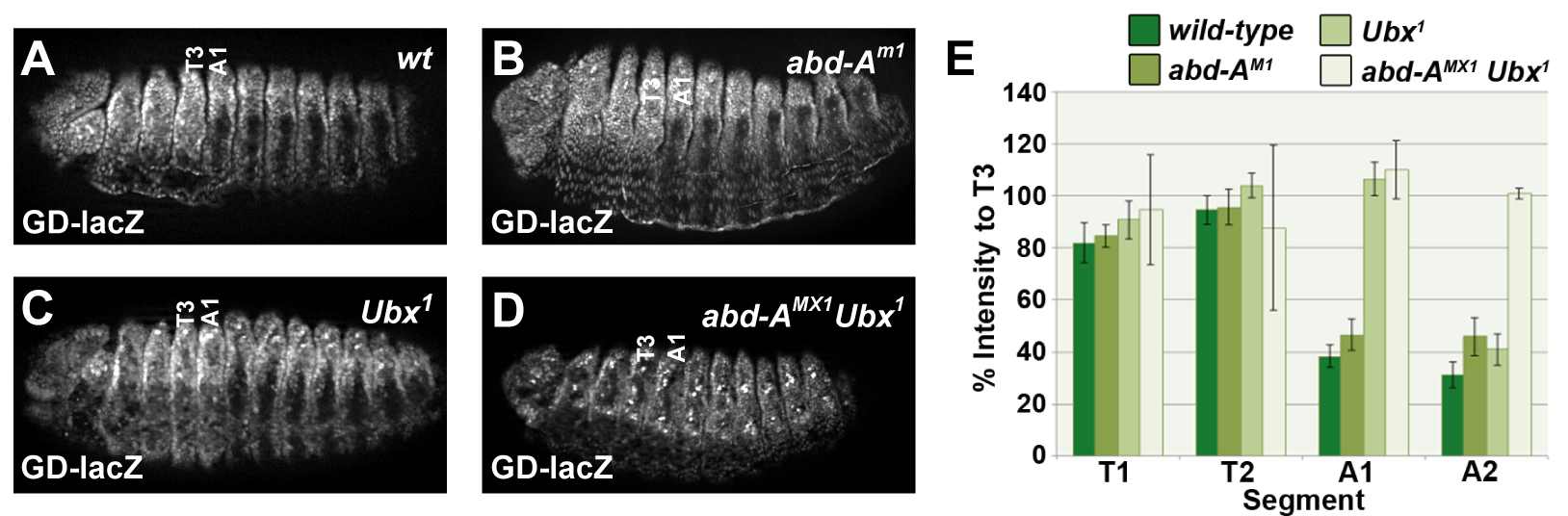

Supplement: S3 Fig — (A). wild-type GD-lacZ embryo. (B) GD-lacZ; abd-AM1 embryo shows that the Ubx Hox factor compensates for loss of Abd-A. (C) A GD-lacZ; UbxMX1 shows that Abd-A compensates for Ubx in segments A2-A7 where it is expressed. (D) A GD-lacZ; abd-AM1, UbxMx12 embryo indicates that the abdominal Hox factors are required for DCRE-mediated repression in the GD-lacZ assay. (E) Quantification of β-gal intensity of panels A-D relative to T3. All images are lateral views of Stage 15 GD-lacZ embryos immunostained for β-gal (white). (TIF) [file pgen.1005981.s003.tif]

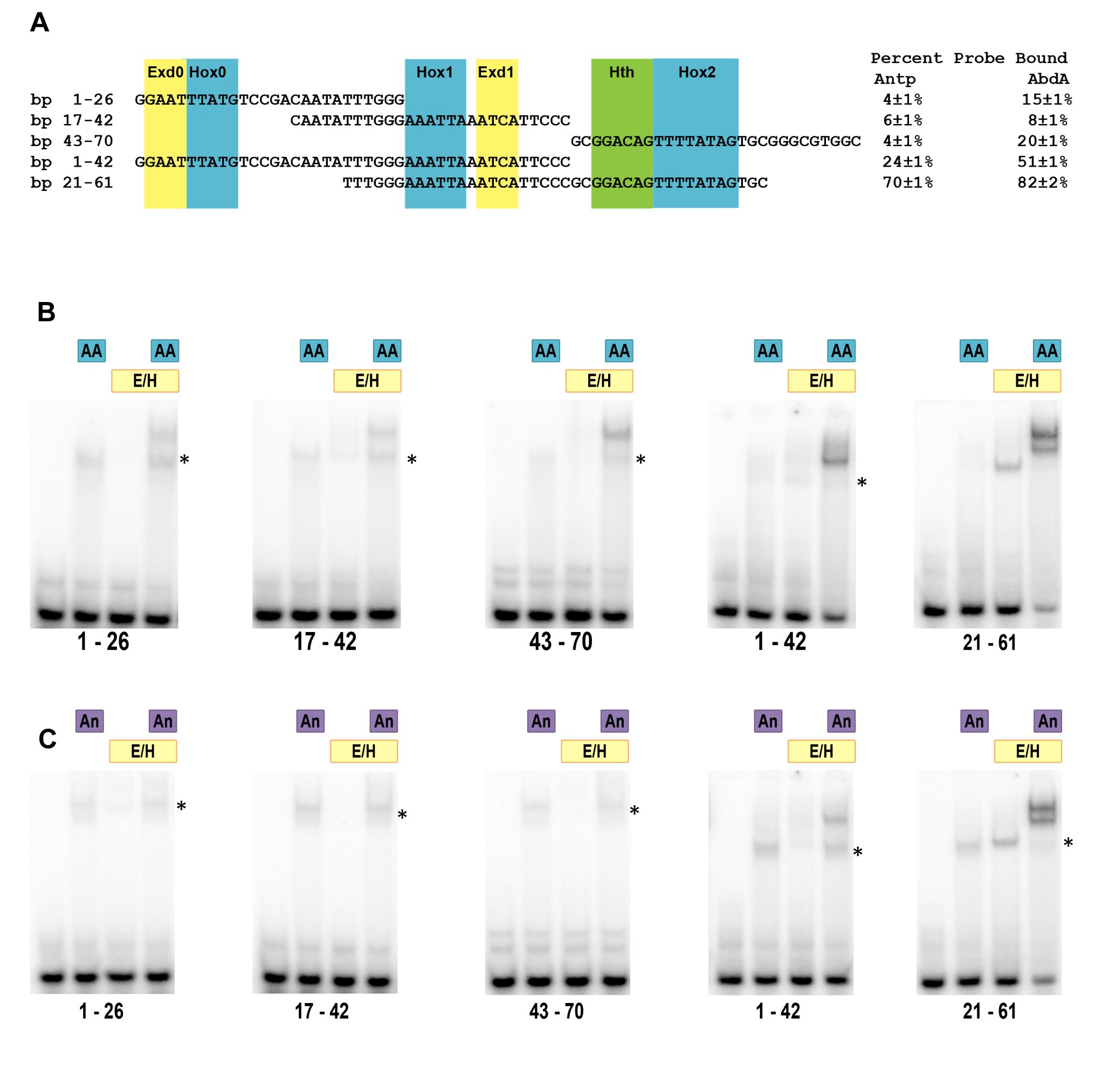

Supplement: S4 Fig — (A) Schematic of the DCRE probes used for EMSAs with Hox, Exd, and Hth sites highlighted. Percent probe bound ± S.E.M. by Hox/Exd/Hth is listed at right for each probe. (B) EMSAs using 25 nM Exd/Hth (E/H, yellow boxes) and 77.5 nM Abd-A (AA blue boxes) on probes containing one or two Hox/cofactor paired sites as listed. (C) EMSAs using 25 nM Exd/Hth and 310 nM Antp (An, magenta boxes) as shown on probes containing one or two Hox/cofactor paired sites. Note that both Abd-A and Antp form complexes on paired Hox/cofactor sites, but Abd-A binds more strongly than Antp. (TIF) [file pgen.1005981.s004.tif]

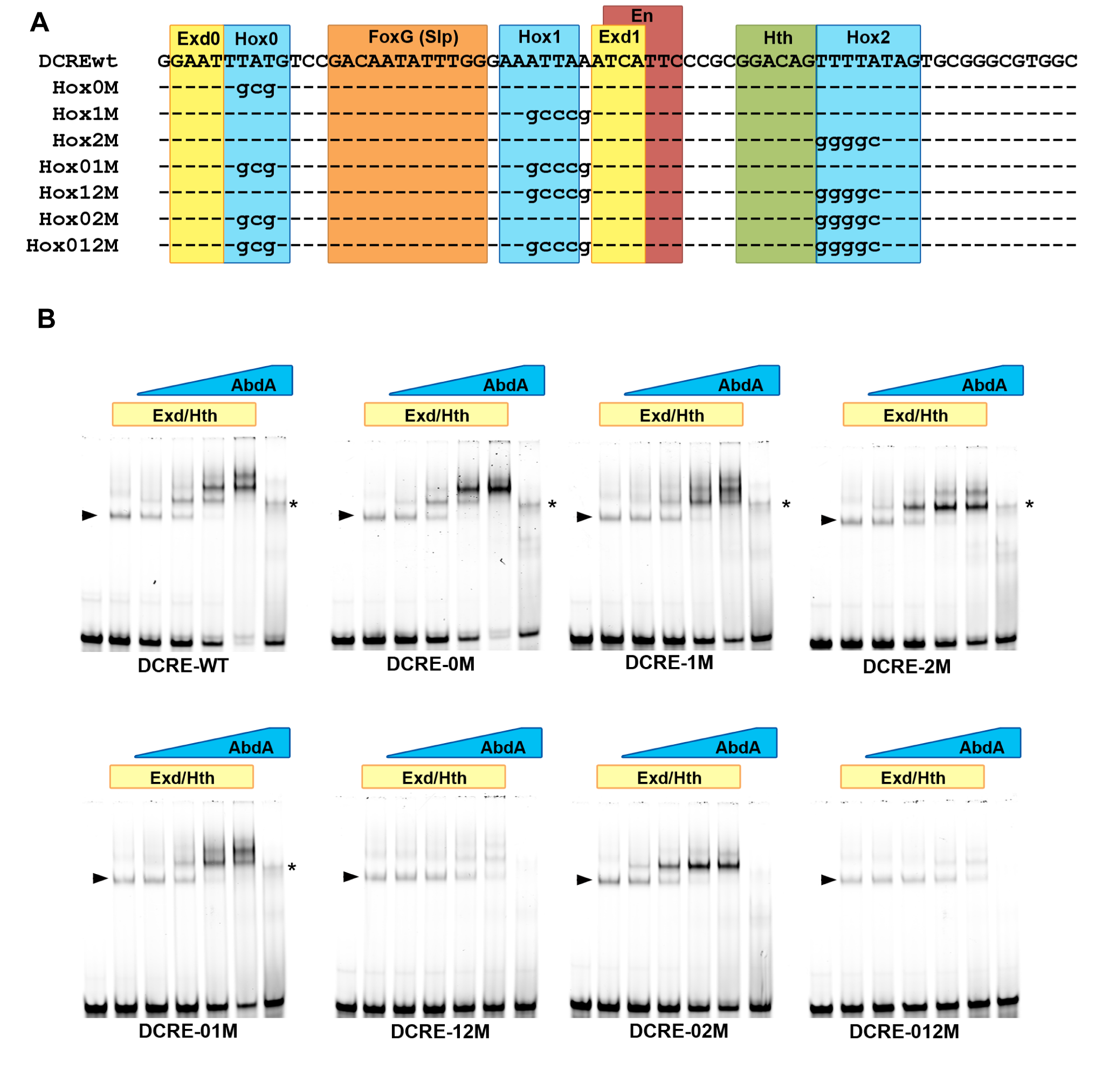

Supplement: S5 Fig — (A) Schematic of the DCRE probes used for EMSAs with Hox, Exd, and Hth sites highlighted. (B) EMSAs performed on the DCRE full-length probes as labeled below each gel. Titration of AbdA protein (concentrations from 37.5 nM to 300 nM) on the DCRE probes as labeled with 25 nM purified Exd/Hth dimer. (TIF) [file pgen.1005981.s005.tif]

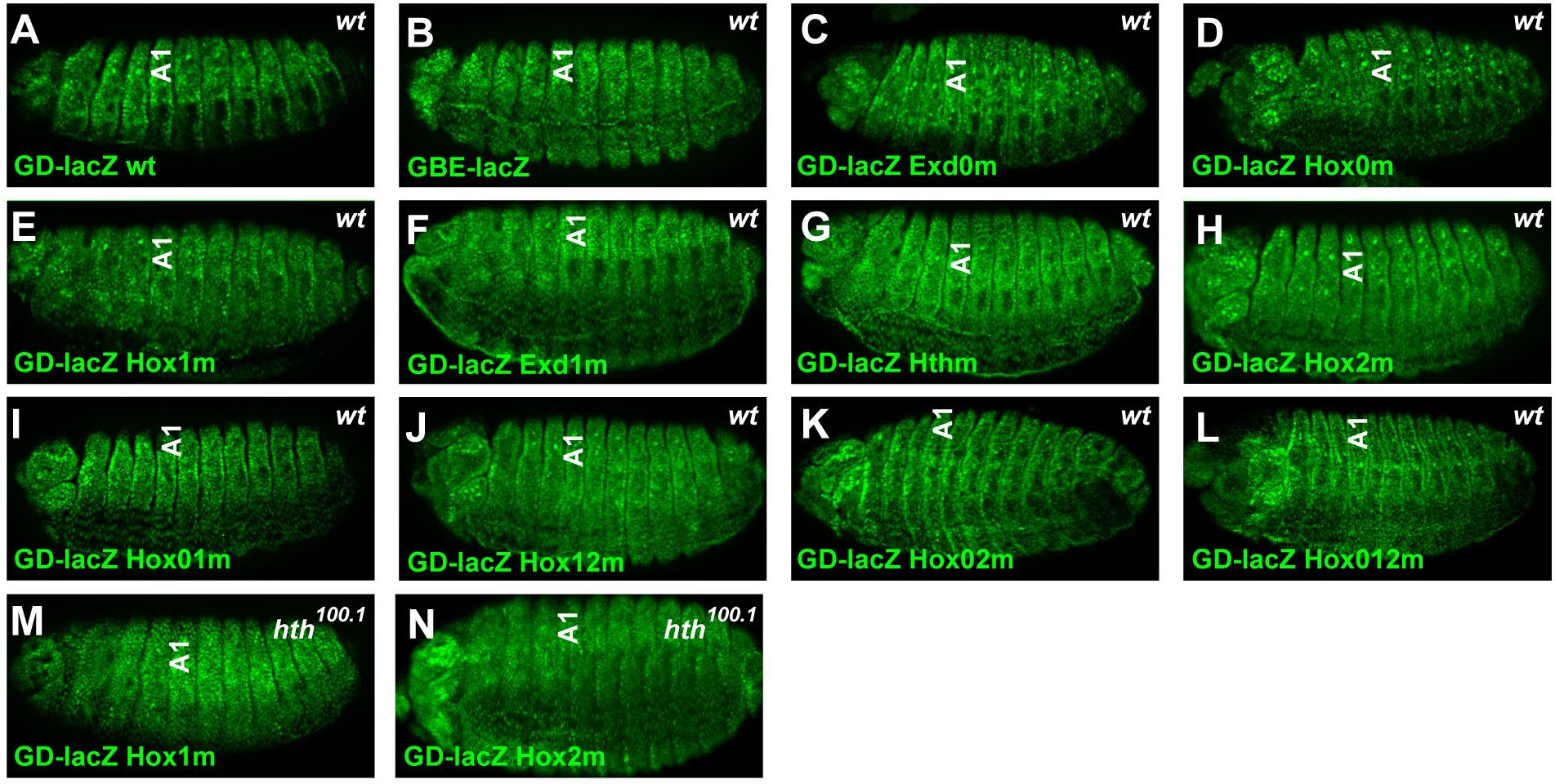

Supplement: S6 Fig — (A-N) Visualization of GD-lacZ reporter in wild-type (wt) (A-L) or hth100.1 (M-N) embryos. For DCRE sequences, see S5 Fig. All images are lateral views of Stage 15 embryos immunostained for β-gal (green). Each GD-lacZ reporter variant is labeled in green in the lower left hand corner of each embryo image, and the genotype of the embryos are labeled in the upper right hand corner. (TIF) [file pgen.1005981.s006.tif]

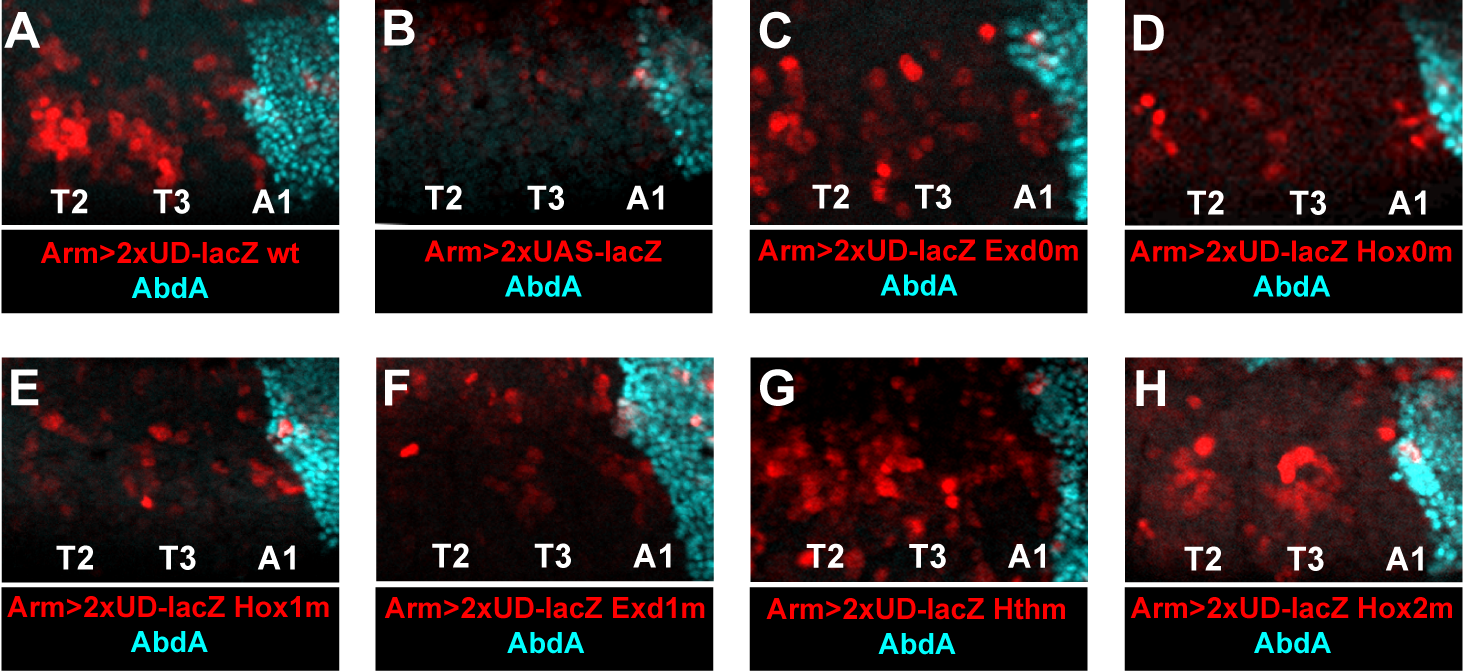

Supplement: S7 Fig — (A-H) Visualization of 2xUD-lacZ reporters in wild-type embryos. For DCRE sequences, see S5 Fig. All images are lateral views of Stage 11 embryos, segments T2, T3, and A1 shown, immunostained for β-gal (red) and AbdA (cyan). Each 2xUD-lacZ reporter variant is labeled below each embryo. (TIF) [file pgen.1005981.s007.tif]

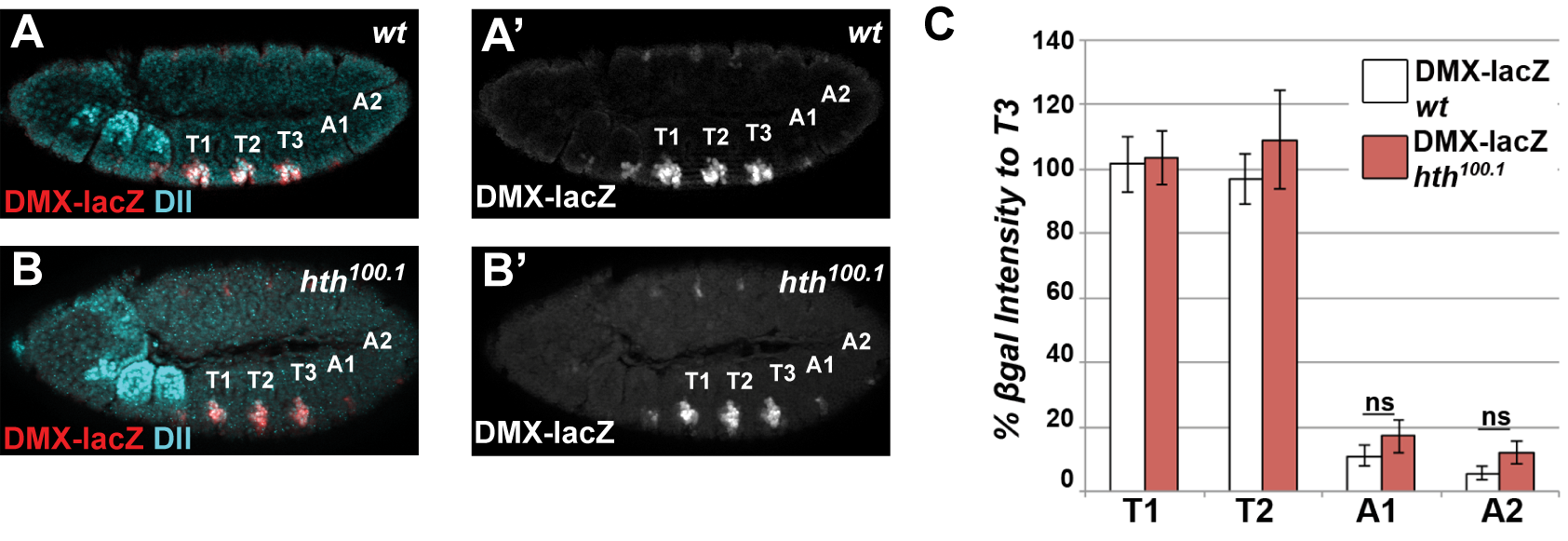

Supplement: S8 Fig — (A-B) Stage 11 DMX-lacZ embryos immunostained for β gal (red) and Dll (cyan), in either a wild type (A) or hth100.1 mutant (B) background demonstrate that Dll and DMX-repression remain normal in absence of the Hth homeodomain. (C) Quantification of DMX-lacZ in wild type and hth100.1 embryos reveals no significant difference in β gal levels of the abdomen or thorax. (TIF) [file pgen.1005981.s008.tif]
